# CRISPR-dCas9 mediated TET1 targeting for selective DNA demethylation at *BRCA1* promoter

**DOI:** 10.18632/oncotarget.10234

**Published:** 2016-06-23

**Authors:** Samrat Roy Choudhury, Yi Cui, Katarzyna Lubecka, Barbara Stefanska, Joseph Irudayaraj

**Affiliations:** ^1^ Department of Agricultural & Biological Engineering, Bindley Bioscience Centre, Purdue University, West Lafayette, IN 47907, USA; ^2^ Department of Nutrition Science, Purdue University, West Lafayette, IN 47907, USA; ^3^ Purdue Centre for Cancer Research, Purdue University, West Lafayette, IN 47907, USA

**Keywords:** CRISPR-dCas9, TET1, BRCA1, DNA demethylation, gene activation

## Abstract

DNA hypermethylation at the promoter of tumour-suppressor genes is tightly correlated with their transcriptional repression and recognized as the hallmark of majority of cancers. Epigenetic silencing of tumour suppressor genes impairs their cellular functions and activates a cascade of events driving cell transformation and cancer progression. Here, we examine site-specific and spatiotemporal alteration in DNA methylation at a target region in *BRCA1* gene promoter, a model tumour suppressor gene. We have developed a programmable CRISPR-Cas9 based demethylase tool containing the deactivated Cas9 (dCas9) fused to the catalytic domain (CD) of Ten-Eleven Translocation (TET) dioxygenase1 (TET1CD). The fusion protein selectively demethylates targeted regions within *BRCA1* promoter as directed by the designed single-guide RNAs (sgRNA), leading to the transcriptional up-regulation of the gene. We also noticed the increment in 5-hydroxymethylation content (5-hmC) at the target DNA site undergoing the most profound demethylation. It confirms the catalytic activity of TET1 in TET1-dCas9 fusion proteins-mediated demethylation at these target sequences. The modular design of the fusion constructs presented here allows for the selective substitution of other chromatin or DNA modifying enzymes and for loci-specific targeting to uncover epigenetic regulatory pathways at gene promoters and other selected genomic regions.

## INTRODUCTION

Epigenetic modifications, including DNA methylation, histone covalent modifications and non-coding RNA mechanisms, have attracted a significant amount of attention for the prevention and treatment of different disorders with cancer at the forefront, mainly due to the inherent reversibility of epigenetic states [1–4]. In particular, aberrations in DNA methylation patterns, including hypermethylation of tumour suppressor genes linked to transcriptional silencing, have been extensively studied and described as hallmarks of nearly all types of cancer [4, 5]. The conversion of cytosines to 5-methylcytosine (5-mC), catalyzed by DNA Methyltransferases (DNMTs) was considered as the stably heritable epigenetic trait until the recognition of acquired demethylation in several physiological processes, such as zygotic epigenetic reprogramming, early embryonic development, somatic cell reprogramming, removal of gene imprinting and development of primordial germ cells [6–10]. These demethylation events were subsequently correlated to the existence and activity of a family of Ten-Eleven Translocation dioxygenase (TET1, TET2, TET3) enzymes, which catalytically convert 5-mC to 5-hmC (5-hydroxymethylcytosine) in presence of 2-oxoglutarate and iron (II) excluding the DNMT actions [11–14]. Moreover, all three TETs have been shown to further oxidize 5-hmC to 5-formylcytosince (5-fC) and 5-carboxylcytosine (5-caC) [15, 16]. These modified cytosines are then frequently subjected to deamination, glycosylase dependent excision or DNA repair mechanisms leading to replacement with unmodified cytosines [17, 18]. These findings collectively suggest that TET enzymes are indirectly but actively involved in the process of DNA demethylation [18].

Given the significant role that has been uncovered for DNA hypermethylation in cancer development, there has been a substantial amount of interest in exploring the role of TET enzymes to be used as an eraser of the aberrant DNA methylation marks and modulate the gene expression. Some recent studies have reported novel approaches, such as fusing TET enzymes to the zinc fingers or transcription activation like repeat elements (TALE) of selective demethylation at the hypermethylated promoter of suppressed or inactive genes [19, 20]. Recently, CRISPR-Cas9 (clustered, regularly interspaced, short palindromic repeat-CRISPR associated protein) mediated engineering tools have gained attention of synthetic biologists for their facile yet efficient endogenous genome editing properties [21]. In particular, catalytically inactive Cas9 (dCas9) in combination with single-guide RNA (sgRNA) are frequently being fused to various transcriptional factors (TFs) such as KRAB-repressors or VP64 activators to respectively inactivate (CRISPRi) or activate (CRISPRa) expression of a given gene [22, 23]. However, CRISPR-Cas9 mediated selective epigenome editing has not yet been extensively practiced. Herein, we used dCas9 fused to the TET1 catalytic domain (TET1CD) and co-treated with various combinations of sgRNA to demonstrate targeted epigenetic editing namely demethylation at the promoter of *BRCA1*, a model tumour suppressor gene.

A plethora of clinical studies have extensively documented occurrence of hypermethylation at the *BRCA1* promoter leading to gene silencing in nonfamilial breast and ovarian cancers [24–26].

On the contrary, overexpression of *BRCA1* was associated with increased apoptosis and inhibition of cell growth in breast and ovarian cancer cells [27, 28]. Silencing of *BRCA1* was linked to increased risk of several types of cancer, which are not limited to breast or ovarian types, but also include cervical, pancreatic, uterine, colon, and prostate cancers [29–31]. These studies cumulatively suggest the demand of targeted approaches to induce *BRCA1* expression in cancer cells and thus achieve possible therapeutic benefits. Herein, we demonstrate active and on demand targeting of TET1CD to the *BRCA1* promoter region using a CRISPR/dCas9 platform in order to decrease DNA methylation, re-activate gene expression and to restore *BRCA1* functional activity in breast and cervical cancer. This approach can be expanded as a tool for targeted demethylation of other tumour suppressor genes that are epigenetically silenced in human cancers.

## RESULTS

### Construction and expression of TET1CD-dCas9 fusion proteins in cells

**Figure 1:**
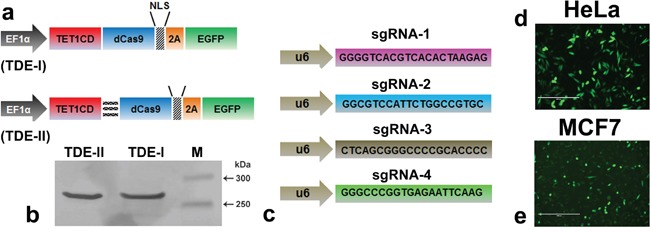
The modular design of CRISPR-dCas9 system for TET1 mediated targeting at *BRCA1* promoter **a.** Schematic illustration of the deactivated Cas9 (dCas9) and TET1 catalytic domain (TET1CD) fusion proteins (TDEs). **b.** Western blot analysis demonstrates production of intact TDE-I and TDE-II (~269 kD) fusion proteins, as observed in lane-1 and lane-2 respectively. **c.** Schematic of single guided RNA sequences, specific to different loci of the *BRCA1* promoter target region (d, e). Representative fluorescent micrographs showing the transfection rate of TDE-I in **d.** HeLa and **e.** MCF7 cells

The constructs were generated by fusing the catalytic domain of TET1 to the N-terminus of deactivated Cas9 tagged with fluorescent reporter EGFP (Figure 1a). We made the fusion proteins either in absence or presence of long linker sequence between TET1CD and dCas9, and defined as TDE-I and TDE-II construct respectively. The transient expression of TDE plasmids in HeLa cells resulted in the full length fusion proteins of ~269 kDa mass (Figure 1b). We also observed the excitation spectra of EGFP in the cell free protein extract of TDE transfected cells using fluorescence spectrometry in both cell lines (Figure S1). TDE-I or TDE-II were co-transfected with different combinations of sequence-specific sgRNAs (Figure 1c). Transient transfection efficiency with TDE-I and TDE-II reached respectively up to 80.8% (Figure 1d) and 84.6% (data not shown) in HeLa cells. In comparison, transfection efficiency with TDE-I and TDE-II reached up to 53.8% (Figure 1e) and 56.2% (data not shown) in MCF7 cells. Per design, upon co-transfection TDE and sgRNAs could be targeted to specific sequences at the *BRCA1* promoter.

### Selective demethylation of *BRCA1* promoter in HeLa cells upon transfection with TET1CD-dCas9 and sgRNAs

**Figure 2:**
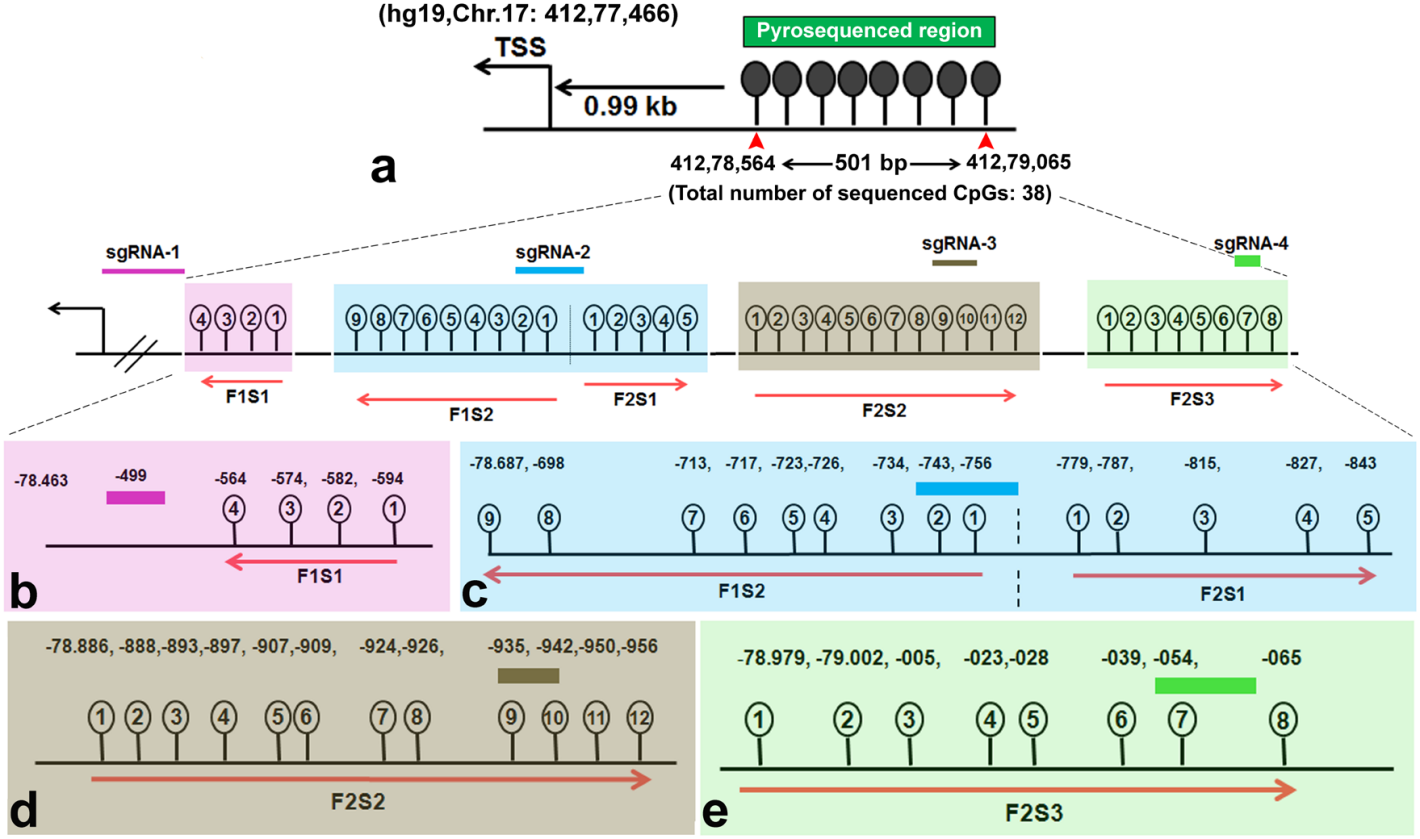
Target *BRCA1* promoter region for loci specific demethylation **a.** Schematic of *BRCA1* promoter region targeted by TET1 using CRISPR-Cas9. The sequenced region is 501 bp long and located within CpG island 0.99 kb upstream from the transcription start site (TSS) (Chr.17:412.78.463). **b.** The binding sites of each sgRNA in the *BRCA1* promoter region and their adjacent CpG sites are depicted in the magnification in lower panel. CpG sites were pyrosequenced with a panel of primers. Four CpG sites adjacent to sgRNA-1 are highlighted in light pink (Fragment #1) and sequenced with primer #1 (F1S1). Nine CpGs adjacent to sgRNA-2 are highlighted in light blue (Fragment #1) and sequenced with primer #2 (F1S2). Five CpGs adjacent to sgRNA-2 are highlighted in light blue (Fragment #2) and sequenced with primer #3 (F2S1). Twelve CpGs adjacent to sgRNA-3 are in dark yellow (Fragment #2) and sequenced with primer #4 (F2S2). Eight CpGs adjacent to sgRNA-4 are in light green (Fragment #2) and sequenced with primer #5 (F2S3). Primer F1S1 and F1S2 sequenced CpG sites in a reverse direction.

Next, we quantitatively determined the extent of targeted demethylation at regions in *BRCA1* promoter using pyrosequencing (please see Figure 2a for the gene map and covered CpG sites). The target promoter region also harbors binding sites for multiple *BRCA1* high-confidence TFs, as identified by the TRANSFAC TFs binding site prediction tool (Biobase). In this section, we report different levels of demethylation at the target CpG sites, which resulted from the combined treatment of sgRNAs with TDE-I or TDE-II in HeLa or MCF7 cells, compared to the TDE transfected cells (served as negative control).

**Figure 3:**
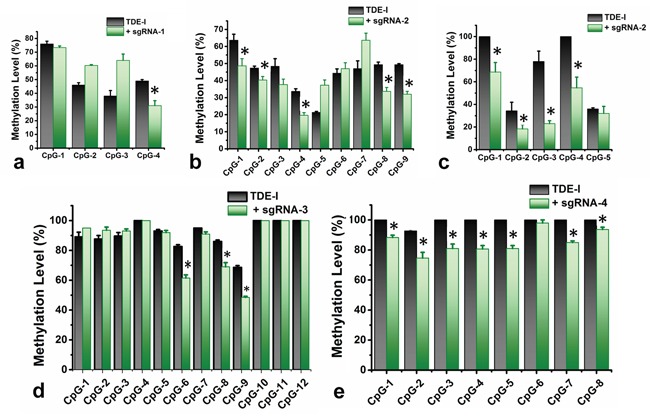
Quantitative determination of DNA demethylation levels at the target CpG sites in *BRCA1* promoter The levels of DNA methylation were determined by pyrosequencing in HeLa cells, treated with TDE-I and different combinations of sgRNAs (in green), compared to only TDE-I treated ones (in black) **a-e.** A *p* value of < 0.05 was considered statistically significant for all the obtained data.

We sequenced methylation level at 38 CpG sites in the selected region (501 bp long) at *BRCA1* promoter. The entire target region was amplified in two fragments (F1 and F2 respectively) and sequenced with a series of primers. For instance, Primer F1S1 was bound to the reverse DNA strand of the target site and sequenced the four CpG sites in a reverse direction, which were within a distance of 80 bp from the sgRNA-1 binding site (Figure 2b). We have observed an 18% reduction in methylation at the CpG-4 site, which was eventually most adjacent to the TDE-I and sgRNA-1 binding site (Figure 3a). However, no or insignificant reduction in methylation was observed at the other three relatively distant CpGs (Figure 3a). Interestingly, ≥ 20% demethylation was observed at all four CpG sites to the 3′-end of the TDE-II and sgRNA-1 binding site (Figure S2a). Primer F1S2 sequenced nine CpG sites in a reverse direction similarly as F1S1. Two of these CpGs (CpG-1 and 2) overlapped with the sgRNA-2 binding site and the remaining seven CpGs were within the distance of 80 bp from the 5′-end of TDEs and sgRNA-2 binding site (Figure 2c). For TDE-I and sgRNA-2, we observed 10-15% reduction in methylation at CpGs1-4, and CpGs 8, 9 (Figure 3b). No significant changes in DNA methylation at those 9 CpGs were detected for a combination of sgRNA-2 and TDE-II (Figure S2b). Primer F2S1 sequenced five CpG sites spanned over 85 bp from 3′-end of the TDEs and sgRNA-2 binding site (Figure 2c). For TDE-I plus sgRNA-2 treated cells, we detected the most profound 31-55% demethylation at CpG-1, 3, and 4. The methylation level was reduced by 16% at CpG-2 (Figure 3c). In contrast, none of these CpG sites were affected by TET1 activity, when cells were treated with combination of TDE-II and sgRNA-2 (Figure S2c). Primer F2S2 sequenced twelve CpG sites, of which eight CpGs (CpG-1 to CpG-8) were spanned over 46 bp from the 5′-end of TDE and sgRNA-3 binding site; two CpGs (CpG-9 and CpG-10) overlapped with the sgRNA-3 binding site, and two CpGs (CpG-11 and CpG-12) were within 10 bp from the 3′-end of sgRNA-3 binding site (Figure 2d). For TDE-I plus sgRNA-3, demethylation was observed at CpGs 6-9, which were located within or the closest to 5′-end of the sgRNA-3 binding site. The strongest decrease in DNA methylation was detected at CpGs 6, 8, and 9 (approximately 20%) (Figure 3d). In contrast, none of the CpG sites were demethylated followed by co-targeting with TDE-II and sgRNA-3 (Figure S2d). Finally, primer F2S3 sequenced eight CpG sites, flanked over 97 bp. Six of those CpG sites (CpG-1 to CpG-6) were within 71 bp from the 5′end of sgRNA-4 binding site, CpG-7 was located within sgRNA-4 binding site, CpG-8 within 10 bp from the 3′-end of the sgRNA-4 binding site (Figure 2e). For TDE-I plus sgRNA-4, we observed various degrees of demethylation at each CpG site (Figure 3e). Similarly, slight decrease was observed for this region in cells transfected with TDE-II plus sgRNA-4 (Figure S2e). In any of the aforementioned sites, dCas9-EGFP plasmids and individual sgRNAs failed to exert any change in the methylation level (data not shown).

Simultaneously, we have co-transfected the cells with the fusion protein containing inactive catalytic domain of TET1 and dCas9 in combination with the best-working sgRNA i.e. sgRNA-2 to determine the accuracy of TET1 mediated demethylation at the target region in *BRCA1* promoter. However, we did not observed any noticeable change in the methylation level followed by this treatment (Figure S5). In addition, we have evaluated the possibility of off-target effects of this CRISPR mediated TET1 targeting by measuring the methylation level at global LINE-1 genomic repeat sequences using the combination of TDE-I and sgRNA2. We however, did not observe any significant changes in any of the CpG sites at the LINE-1 repeat sequence (Figure S6). These multiple pieces of evidence hence strongly suggest the CRISPR mediated site specific demethylation at *BRCA1* promoter by the TET1CD from the TDE fusion proteins.

### *BRCA1* expression in HeLa cells upon transfection with TET1CD-dCas9 and sgRNAs

**Figure 4:**
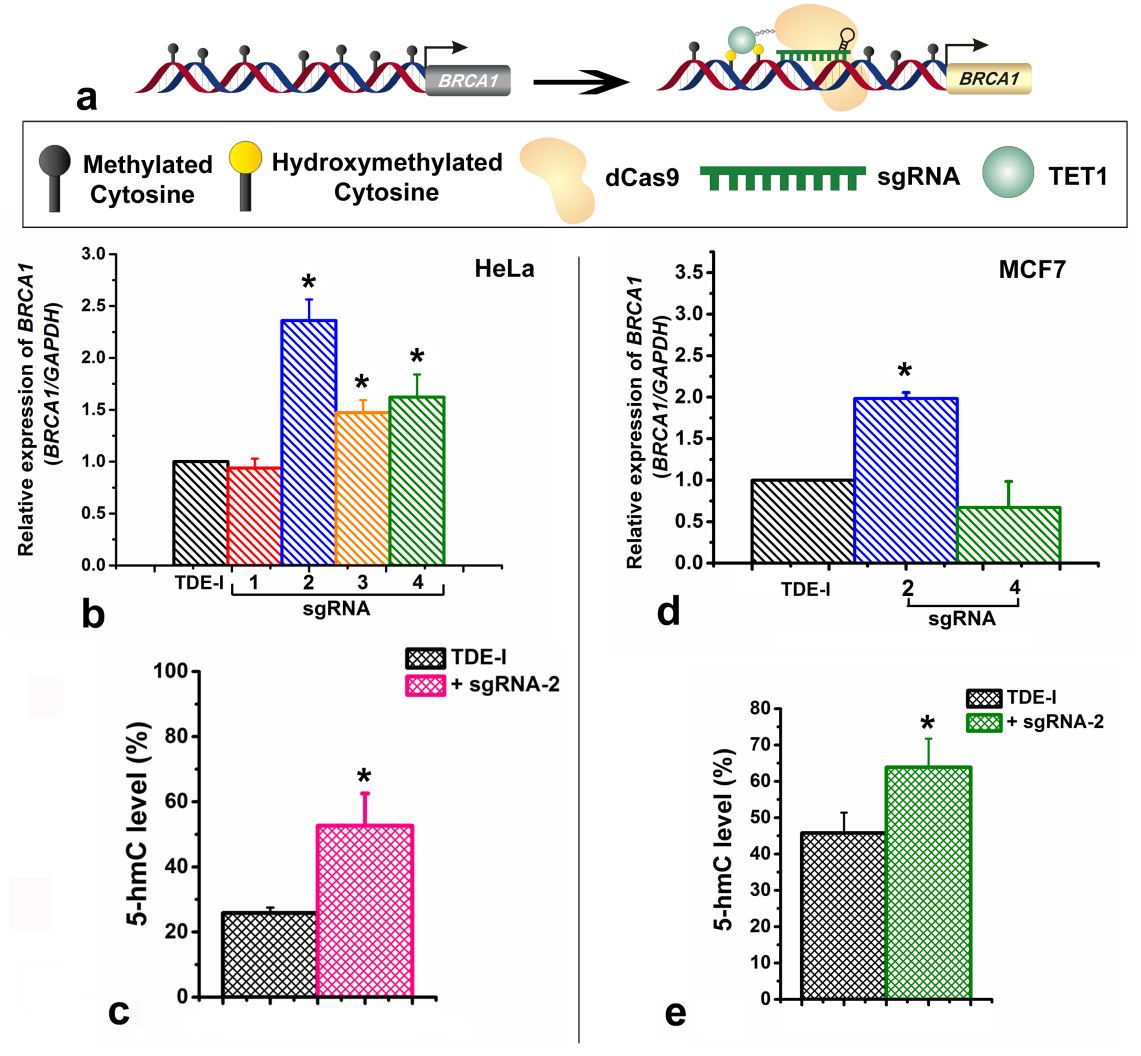
Effects of targeting of TET1 to BRCA1 promoter on gene expression and on enrichment with 5-hydroxymethylation (5-hmC) **a.** Schematic representation showing the up-regulation of BRCA1 gene expression through loci specific hydroxymethylation (demethylation) by the TET1-dCas9 fusion protein. **b.** BRCA1 gene expression after treatment of HeLa cells with TDE-I and different sgRNAs as determined by qPCR. **c.** Enrichment of BRCA1 promoter with 5-hmC after transfection of HeLa cells with TDE-I and sgRNA-2. **d.** BRCA1 gene expression after treatment of MCF-7 cells with TDE-I and sgRNA-2. **e.** Enrichment of BRCA1 promoter with 5-hmC after transfection of MCF-7 cells with TDE-I and sgRNA-2.

We determined the possible effect of CRISPR/Cas9-TET1 mediated demethylation at the *BRCA1* promoter on the transcriptional activity of the gene, using qPCR (Figure 4a). We observed the highest degree of *BRCA1* up-regulation for TDE-I and sgRNA-2 (Figure 4b). This was associated with the highest extent of *BRCA1* demethylation (Figure 3b and 3c). Less profound but consistent demethylation across multiple CpG sites was detected for TDE-I and sgRNA-4 (Figure 3e) as well as for TDE-I and sgRNA-3 (Figure 3d), which correlated with increase in *BRCA1* expression (Figure 4b). Interestingly, the extent of demethylation was reflected in the degree of gene up-regulation. On the other hand, insignificant or no increase in *BRCA1* expression was observed when HeLa cells were transfected with TDE-I and sgRNA-1 or TDE-II and different sgRNAs (Figure S4). Importantly, lack of effects on *BRCA1* expression was associated with no changes in DNA methylation, except TDE-II and sgRNA-1. For the latter combination, despite demethylation of *BRCA1* promoter (Figure S2a), no increase in gene expression was detected (Figure S4).

### *BRCA1* promoter methylation and gene expression in MCF7 cells transfected with TDE-I and sgRNA-2 or sgRNA-4

As the highest demethylation of *BRCA1* promoter coinciding with the highest increase in *BRCA1* expression was detected for combinations of TDE-I with sgRNA-2 or sgRNA-4, we assessed the effects of these combinations in another cell line, namely MCF7 breast cancer cells. In MCF7, we did not observe any significant demethylation at any of the nine CpG sites sequenced with F1S2 primer (TDE-I and sgRNA-2) (Figure S3a). In a fragment sequenced with F2S1 (TDE-I and sgRNA-2), we observed demethylation of 4% at CpG-2 and CpG-4, 7% at CpG-3, and 13% at CpG-5 (Figure S3b). As expected, demethylation caused by TDE-I and sgRNA-2 was linked to *BRCA1* up-regulation (Figure 4d). Transfection with TDE-I and sgRNA-4 however, did not lead to any significant changes in either methylation (Figure S3c) or gene expression of *BRCA1* (Figure 4d). Less profound effects observed in MCF-7 cells compared with HeLa cells may be a result of lower efficiency of transfection as shown in Figure 1d and 1e.

### Locus specific hydroxymethylation at the CRISPR/dCas9-TET1 target sites

Since, TET1 results in demethylation usually through hydroxylation of the 5-mC, we further assessed the level of 5-hmC at CRISPR-TET1 targeted sites at the *BRCA1* promoter. We assessed the locus-specific changes in 5-hmC content at the adjacent binding sites of TDE-I plus sgRNA-2, since this combination leads to the most profound demethylation and gene activation in both tested cell line. We have observed an increase in 5-hmC level by ~25% at the sequence adjacent to the TDE-I and sgRNA-2 binding site in HeLa cells (Figure 4c). In addition, we observed ~15% increase in 5-hmC level at the same region in MCF7 cells (Figure 4e). The elevated levels of 5-hmC in both the cell line correlate with DNA demethylation, which corroborates the fact that TET1CD was catalytically active in our targeted fusion proteins (TDEs) and leads to the site specific demethylation.

### Up-regulation of BRCA1 through targeted demethylation inhibits cell growth

**Figure 5:**
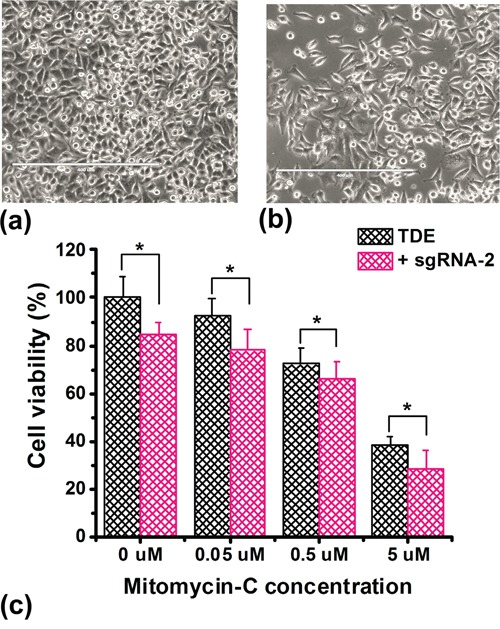
Overexpression of *BRCA1* inhibits cell proliferation The effect of co-treatment of HeLa cells with TDE-I and sgRNA-2 on cell viability under Mitomycin-C (MMC) stress. Control and transfected cells were treated with different concentrations of Mitomycin-C for 72 hr followed by MTT assay. The bars represent the mean value of cell viability of the biological replicates (n=8) and SD between the experiments. A *p value* of < 0.05 was considered statistically significant for all the obtained data.

In order to evaluate whether TET1CD-dCas9-mediated increase in *BRCA1* expression has any biological impact, we measured cell viability upon treatment of cells under the Mitomycin-C (MMC) stress with TDE-I and sgRNA-2. HeLa cells were more resistant to MMC treatment and ~ 40% of cell viability was still observed at 5 μM concentration. In contrast, MCF7 cells were more sensitive to MMC and treatment with concentrations higher than 0.5 μM caused significant cell death. In HeLa cells, *BRCA1* up-regulation mediated by treatment with TDE-I and sgRNA-2 enhanced the inhibitory effect of MMC on cell growth (Figure 5a-5c). In MCF7 cells, we observed a slight but non-significant reduction in cell viability, followed by treatment with TDE-I plus sgRNA-2 compared to control cells treated with TDE-I alone in the presence of 0.5 μM MMC (data not shown).

## DISCUSSION AND CONCLUSIONS

The recent emergence of CRISPR-Cas9 system allows for precise and stable editing of the genome or epigenome at any guided RNA mediated target site and by fusing the Cas9 endonuclease with any transcription regulators such as VP64 or KRAB domain or with any epigenetic enzymes [32]. CRISPR tools use sgRNAs that specifically bind to the target DNA sequence, and serve as a scaffold to recruit CRISPR dependent Cas9 nuclease [33]. In addition, sgRNA-dCas9 combination may acts as a molecular homing device for the secondary effector proteins such as methyltransferases or demethylases to efficiently alter the epigenetic status of the target DNA or histone covalent modifications [34].

Customized sgRNAs in combination with the dCas9-TET1CD fusion proteins (TDEs), developed here are demonstrated to efficiently alter methylation status of the target sequences of *BRCA1* gene promoter. Insignificant off-target methylation effects, as observed at the LINE-1 repeat sequences further confirms the target specificity of the tools, developed in the present study (Figure S6). The increase in locus specific 5-hmC (Figure 4c and 4e) and concomitant reduction in methylation level at the target sequenced sites (Figure 3 and Figure S3) also suggest the biocatalytic efficacy of TET1 in TDE-I. This is more convincing because the combinatorial treatment with sgRNA-2 and inactive TET1 fusion protein resulted in no noticeable changes at the methylation level (Figure S5) or gene expression (data not shown). Our aim was also to optimize the length of a linker sequence between the functional domain of dCas9 and TET1CD, which would produce the most profound loci-specific demethylation effects. Thus, we constructed two fusion proteins, TDE-I without a linker sequence and TDE-II that contained a long linker sequence. Our expectation was that a long linker sequence would allow TET1 to move more freely around the site of its binding and presumably affect additional CpG sites neighbouring the target sites. Interestingly, TDE-I fusion protein that was designed without a linker worked better in terms of inducing demethylation at the target CpG sites (Figure 3 and Figure S3). The extent of demethylation were negligible for the TDE-II treated cells, compared to TDE-I (Figure S2). Hence, this reduced efficacy of TDE-II can be attributed to the presence of the long linker sequence, which might cause TET1CD to move around the DNA strands with more flexibility missing the ample access to the target CpG sites.

DNA hypermethylation of gene promoters and other gene regulatory regions has been frequently reported in conjunction with silenced/downregulated gene expression or as epigenetic biomarkers of different types of cancer [1, 3, 35]. Methylation-mediated silencing of transcription was demonstrated in numerous studies predominantly for tumour suppressor genes. Induced demethylation of those regions in tumour suppressor genes found to be tightly linked to the increased gene expression [36, 37]. One of classical examples of tumour suppressor genes silenced through DNA methylation is *BRCA1* [24, 38]. Demethylating agent, 5-aza-deoxycytidine, decreased *BRCA1* promoter methylation resulting in gene-re-activation [39]. Hence, based on the experimental findings, we can attribute the increase in *BRCA1* gene expression to the selective demethylation at the promoter region in our experimental settings. The highest level of demethylation was observed at CpG sites proximal to the sgRNA-2 and TDE-I binding site, which correlated with the most profound increase in *BRCA1* expression. A similar correlation between demethylation and gene expression was obtained for the combination of sgRNA-4 or sgRNA-3 with TDE-I. This would suggest the relevance of regions targeted with sgRNA-2, sgRNA-3, and sgRNA-4 in regulation of *BRCA1* transcription. Methylation may disrupt binding of transcription factors to regulatory regions of *BRCA1* as previously suggested [39, 40]. Indeed, regions targeted with sgRNA-2, sgRNA-3, and sgRNA-4 are enriched with multiple transcription factors, including Sp1 and Egr-1, as predicted using TransFac. Sp1 and Egr-1 are activators of *BRCA1* [41, 42] and their binding to DNA is known to be impaired by DNA methylation [43, 44]. Hence the reversal of methylation, i.e., demethylation, can be logically considered to facilitate transcription factor binding to DNA and subsequent initiation of transcription. In contrast, insignificant changes in DNA methylation were observed followed by the co-treatment with sgRNAs and TDE-II, except the combination with sgRNA-1. Despite decrease in methylation at the CpGs adjacent to the sgRNA-1 and TDE-II binding site, we did not observe *BRCA1* overexpression in the treated cell population. It is possible that this region does not play a regulatory role in *BRCA1* transcriptional activity. Another explanation may be that the long linker sequence between the functional domain of dCas9 and TET1CD in TDE-II enables demethylation of CpG sites but masks the sequence making it unrecognizable for transcription factors. As a result, *BRCA1* transcription cannot be activated. Strikingly, CpG sites targeted by sgRNA-1 are demethylated only when we use TDE-II that contains a long linker sequence. Short linker containing fusion protein, TDE-I, does not induce demethylation of those CpGs. Of note, binding of sgRNA-1 occurs 64 bp apart from the first targeted CpG site (Figure 2b), contrary to other sgRNAs where some of the targeted CpG sites overlap with sgRNA binding. Thus, the location of sgRNA binding relative to CpG sites may affect the efficiency of TET1 activity. In the context of demethylation at the target sites, we also observed occasional increase in methylation level, such as CpG-5 or CpG-7 at the sgRNA-2 and TDE-I target site (Figure 3b). These unexpected changes in methylation level could be explanined as the putative binding of certain methylation binding proteins (MBDs) to the TET induced hydroxymethylated CpGs at the *BRCA1* promoter. Previous studies showed that UHRF1 (ubiquitin-like, containing PHD and RING finger domains); a MBD family protein, can bind to the hydroxymethylated sites with similar affinity to the methylated sites and may recruit DNMT3A or 3B to create de-novo methylation and inhibit TET activity [45–47]. Moreover, UHRF1-mediated increase in methylation has also been reported as a causal factor in epigenetic silencing of the *BRCA1* gene in sporadic breast cancer [48]. It is possible that CRISPR targeted TET fusion proteins induce the binding of UHRF1 or other putative MBDs to the target CpG sites at the *BRCA1* promoter, to preserve the methylation status. Nonetheless, we have observed significant increase in the *BRCA1* expression, which suggest that the CRISPR targeted TET1 still might efficiently work, despite these practical challenges.

We observed differences in the extent of demethylation at target CpG sites between HeLa and MCF7 cells. DNA demethylation was lower in MCF7 in comparison to the HeLa. This can be attributed to the differences in transfection efficiency in the two cell lines. Transfection was successful in 85% of HeLa cells while only 55% of MCF7 cells were positively stained with EGFP after treatment with TDEs and sgRNAs (Figure 1). Nonetheless, the highest extent of demethylation and overexpression of *BRCA1* was detected for combination of TDE-I and sgRNA-2 in both cell lines. Thus, we can conclude that selective demethylation at the target CpG sites could be possible through targeting the optimal combination of sgRNAs and TDE fusion proteins.

Finally, to validate biological relevance of TET1CD-dCAs9-mediated *BRCA1* overexpression, we assessed the effects of transfection with TDEs and sgRNAs on cell growth in both tested cell lines. The significant reduction in cell growth followed by the treatment with TDE-I and sgRNA-2 suggested that induced increase in *BRCA1* expression have profound inhibitory role against cell viability either under or without stress with chemotherapeutic agents. The inhibitory effects on cell growth were again more profound in HeLa cell, compared with MCF7 cells, which may result from differences in transfection efficiency. To successfully use this current set of CRISPR tools, the best combination of sgRNAs and fusion proteins need to be accompanied by efficient methods of transfection. The latter may require further optimization possibly in conjunction with cell sorting procedures for the best desired result in cell lines that are difficult to transfect.

In conclusion, we have achieved site specific demethylation at targeted loci of *BRCA1* gene promoter by fusing TET1 catalytic domain to the CRISPR-dCas9 system. We showed that lack of a long linker sequence between the functional domain of dCas9 and TET1CD produces the most profound loci-specific demethylation effects. Similarly, location of sgRNA binding site relative to targeted CpG sites may affect the extent of demethylation. Selective epigenetic modifications also resulted in increased gene expression and biological activity of the selected gene. This proof of concept could be applicable for rapid and robust demethylation of any DNA sequence when using specific sgRNAs. Finally, the epigenome editing tools developed here also allows for the selective substitution of other epigenome modifiers such as methyltransferases or demethylases for loci specific targeting to uncover epigenetic regulatory pathways of any gene of biochemical or biomedical importance.

## MATERIALS AND METHODS

### Preparation of the TET1CD- dCas9-EGFP fusion protein

The fusion protein of TET1 catalytic domain (TET1CD), and dCas9 was generated by sequentially assembling the coding sequences of the desired proteins using standard restriction enzyme digest and ligation method. The source plasmids of dCas9 (#51023 from Bo Huang and Stanley Qi lab), and TET1 (#49792 from Anjana Rao lab) were obtained from the Addgene plasmid repository (https://www.addgene.org/). In addition, a fusion protein of only dCas9-EGFP was used as a negative control to the catalytic activity of TET1. Inserts were then amplified by Polymerase Chain Reaction (PCR) from the respective source plasmids with desired restriction sites flanking on either side of the amplicons with CloneAmpHiFi PCR Premix (639298, Clontech Laboratories Inc.). The PCR reaction was carried out as specified by the manufacturer for the template DNA concentration >100 ng with 35 cycles of amplification. Detail of the PCR amplification and sequencing primers have been summarized in Table S1. The inserts were incorporated into the ‘pAAV_EF1α_WPRE_hGHpA’ backbone of mammalian expression vector (plasmid #47457, from Zhang F, Addgene). The original vector was sequentially digested with Acc651 and BamHI to incorporate TET1CD, and BamHI and XbaI to incorporate dCas9. Incorporation of TET1CD and dCas9 into the vector has replaced the CRY2PHR_NLS-VP64 of the original plasmid. The final fusion protein hence formed was in the frame of pAAV_EF1α_TET1CD-dCas9-NLS-2A-GFP_WPRE_hGHpA. Two fusion protein constructs were made by using without or with a long linker sequence between the TET1CD and dCas9 functional domains and defined respectively as TDE-I or TDE-II constructs. PCR amplified inserts and the vector template were digested with restriction endonucleases followed by gel purification using the QIAEX II gel extraction kit (20021, QIAGEN). Purified vector and inserts thus made were ligated along with requisite amount of T4 DNA ligase buffer and enzyme system (M0202S, New England Biolabs) and kept at room temperature for 15 min. The ligated product was then transformed into the stellar competent cells (PT5056-2, Clonetech Laboratories Inc.) and plated out on an Ampicillin (Amp) supplemented LB agar plate. Suitable clones were propagated in LB-Amp media and the plasmids were extracted with QIAprep Spin Miniprep Kit (27104, QIAGEN). The full length nucleotide sequence of the fusion proteins can be found in the Supplementary Information (Supplementary Sequence-1 and 2). The fusion protein was sequenced against a panel of primers as summarized in Table S1. In addition, we have generated fusion protein of dCas9 with inactive TET1CD for the negative control experiments. The preparation of inactive TET1CD-dCas9 fusion protein, PCR primers (Table S2), and the full nucleotide sequence (Supplementary Sequence-3) of the fusion protein can be found in the Supplementary Information.

### Detection of TET1CD-dCas9 fusion protein

The total protein was extracted from the cells, individually or co-transfected with TDE plasmids and sgRNAs respectively, and the excitation spectra of the EGFP fluorescence of the fusion proteins were recorded using the fluorometer (Cary Eclipse, Agilent Technologies). Expression of the fusion proteins in HeLa cells were confirmed with western blot. For performing the western blot, cells were rinsed twice with ice-cold PBS followed by lysis with M-PER mammalian protein extraction reagent supplemented with Halt protease inhibitor cocktail (Thermo Scientific). The concentration of the extracted proteins was then determined with Coomassie Plus (Bradford assay kit; Pierce). Twenty micrograms of the extracted proteins were loaded per lane onto a 4% to 15% polyacrylamide gel (Bio-rad, USA) for electrophoresis and electro-transferred to a nitrocellulose membrane (Biorad). The membrane was then blocked in 5% nonfat dry milk in (TBS-T) Tris-buffered-saline-Tween buffer (10mM Tris (pH 8.0), 140 mMNaCl, 0.1% Tween 20) overnight at 4°C, followed by incubation with rabbit polyclonal anti-EGFP antibody (ab290; Abcam) at 1:1000 dilution overnight in 5% milk-TBS-T. After washing with fresh TBS-T for three times, the membrane was incubated with rabbit secondary antibody conjugated to AlexaFluor 680 (ab150077; Thermo-Scientific) at 1:2000 dilutions in the blocking buffer for 1 h at room temperature. The membrane was re-washed with TBS-T for three times and scanned using a Li-Cor Odyssey scanner (CLx) system.

### Designing of the sgRNA

Four sgRNA strings were designed as PCR cassettes (501 bp) with U6 promoter (Life Technologies, USA), which bind to four different specific sequences in the *BRCA1* promoter. For subsequent uses, the sgRNAs were amplified with a set of primer, which are as follows: Forward: 5′-GGCCTATTGGTTAAAAAATGAGCTG-3′ and Reverse: 5′-GAGCGGATAACAATTTCACACAGGA-3′. PCR amplicons of the sgRNA cassettes were then purified through gel-extraction (20021, QIAGEN). Full length sequence of the sgRNA PCR cassettes and their corresponding binding sites has been described in the Supplementary Information (Supplementary Sequence-4-9).

### Cell culture and transfection

HeLa or MCF7 cells were seeded at the density of 0.7 × 10^6^ and cultured at 37°C with 5% CO_2_ in presence of DMEM/F-12 supplemented with 10% FBS, 1% glutamax and 1% penicillin-streptomycin (Life Technologies). Cells were co-transfected with TET1-dCas9 plasmid plus sgRNA strings using Lipofectamine LTX (Life Technologies) system according to the manufacturer's instruction. Briefly, 70-80% confluent cells in 35 mm plates were co-transfected with 2.5 μg of TET1-dCas9 plasmid and 1 μg of sgRNA strings. Cells individually transfected with dCas9 plasmids were considered as the negative control. Transfection efficiency of the plasmids was assessed with microscopic analyses. The fluorescence intensities of transfected cells were examined under fluorescence microscope (EVOS FL cell imaging system, Life Technologies), 24 h after transfection to assess the expression of the EGFP fluorescence from the TDE fusion proteins. During imaging, cells were incubated with HBSS buffer (pH adjusted to 7.4 with 2 M NaOH).

### Bisulfite converted PCR (BSP-PCR) and pyrosequencing analyses

600 ng of genomic DNA, isolated from the replicates of individually-transfected (with TDE-I or TDE-II plasmids as a control) or co-transfected (with TDE-I or TDE-II plasmids and combination of sgRNAs) cells after 24 h. of transfection. DNA samples were bisulfite converted with EZ DNA methylation kit (D5001; Zymo Research). The bisulfite converted DNA was PCR amplified using two sets of biotinylated primers (fragment F1 and F2), which encompassed the entire target region in the *BRCA1* promoter. The PCR amplification was carried out using PyroMark PCR kit (978703; QIAGEN) as per manufacturer's instruction for 25 μl of reaction volume. The obtained PCR products were then subjected to pyrosequencing (PyroMark Q24 system; QIAGEN) using four sequence specific primers. Sequencing primers F1S1 and F1S2 covered CpGs within fragment F1 and sequencing primers F2S1, F2S2, and F2S3 sequenced fragment F2. The covered CpG sites, corresponding to each of the sequencing primers have been summarized in Table S3. Data were analysed using PyroMarkTMQ24 software.

### *BRCA1* expression analysis by qPCR

The mRNA expression of *BRCA1* was evaluated in comparison to the endogenous control *GAPDH*, from the cells treated with different combinations of sgRNAs and TDEs compared to the individually transfected (TDEs) cells. Primer sequence for *GAPDH* and *BRCA1* has been summarized in Table S4. Briefly, the total RNA was extracted from the 24 h. post-treated cells using RNeasy Mini Kit (74104, QIAGEN, USA) and converted to the c-DNA templates using Quantitect Reverse Transcript PCR (205311, QIAGEN, USA). The change in fold expression of *BRCA1* was then determined in SYBR green master mix (Life Technologies, USA), using quantitative real-time PCR (qPCR) (StepOnePlus Real-Time PCR Systems; v 2.0 Applied Biosystems, USA). Amplification conditions were 95°C for 1 min, followed by 40 cycles at 95°C for 15 sec and 60°C for 1 min. Each sample was assayed in triplicate.

### Detection of locus specific 5-hydroxymethylcytosine (5-hmC)

To determine differential demethylation level at the *BRCA1* promoter through TET1 activity, we continued to assess the sequence-specific detection of 5-hmC at the sites, adjacent to TDEs and sgRNAs binding. Detection of sequence specific 5-hmC were carried out using the Quest 5-hmC Detection Kit-Lite (D5415, Zymo Research, USA) as per manufacturer's instructions. Briefly, genomic DNA was extracted from the cells, transfected individually with TDE or co-transfected with TDE plus sgRNAs. 500 ng of genomic DNA was then treated with 5-hmC gluosyltransferase (GT), which specifically adds a glucose moiety to the 5-hmC sites and makes it resistant to the digestion with any glucosyl-5hmC sensitive restriction endonucleases (GlaI in this case). In contrast, GlaI is able to cleave at cytosine, 5-mC, and non-glucosylated 5-hmC sites. To identify the amount of 5-hmC at the target site in *BRCA1* promoter, 50 ng of DNA obtained from the aforesaid procedure were amplified using qPCR with a set of primers, designed to cover the adjacent glucosylated 5-hmC sites. Primer sequences and the corresponding covered sites are summarized Table S5. The qPCR amplification conditions are same as mentioned for the detection of *BRCA1* expression.

### Cell viability assay

MTT (3-(4, 5-dimethylthiazol-2-yl)-2, 5-diphenyl-2H-tetrazolium bromide) based cell viability assay was performed to assess the cell-proliferation in the TDE plus sgRNA treated cells. Co-transfected cells (both HeLa and MCF7) were seeded onto 96-well plates at a density of 5,000 cells/well. After 24 h. cells were treated with five different concentrations (0, 0.05, 0.5, 5, and 50 μM) of mitomycin C (MMC; Cayman Chemicals, USA). After 72 h. of continuous drug exposure, cells were rinsed with 1X PBS followed by incubation with the MTT reagent (20 μl of 5 mg/mL) for 4 h. at 37°C in the presence of CO_2_. The developed formazan was dissolved in 100 μL of acidic isopropanol and the optical density was recorded on a microplate reader (Spectra max plus 384, Molecular Devices, USA) at 570 nm to determine the percentage of cell viability. Absorbance values were normalized to the control wells with the culture medium alone.

### Statistical analysis

We have used a two-way student's *t-*test to determine the statistically significant difference between the control and treated groups. A *p* value < 0.05 was considered to be significant.

## SUPPLEMENTARY DATA




