# Supplementary material for: CRISPR-dCas9 mediated TET1 targeting for selective DNA demethylation at BRCA1 promoter
Source: Oncotarget. 2016 Jun 23;7(29):46545–56. doi: 10.18632/oncotarget.10234 (PMC5216816; doi:10.18632/oncotarget.10234)
Supplement: Supplementary file 1 [file oncotarget-07-46545-s001.pdf]

CRISPR-dCas9 mediated TET1 targeting for selective DNA demethylation at *BRCA1* promoter

SUPPLEMENTARY DATA

See Supplementary Sequence File: 1

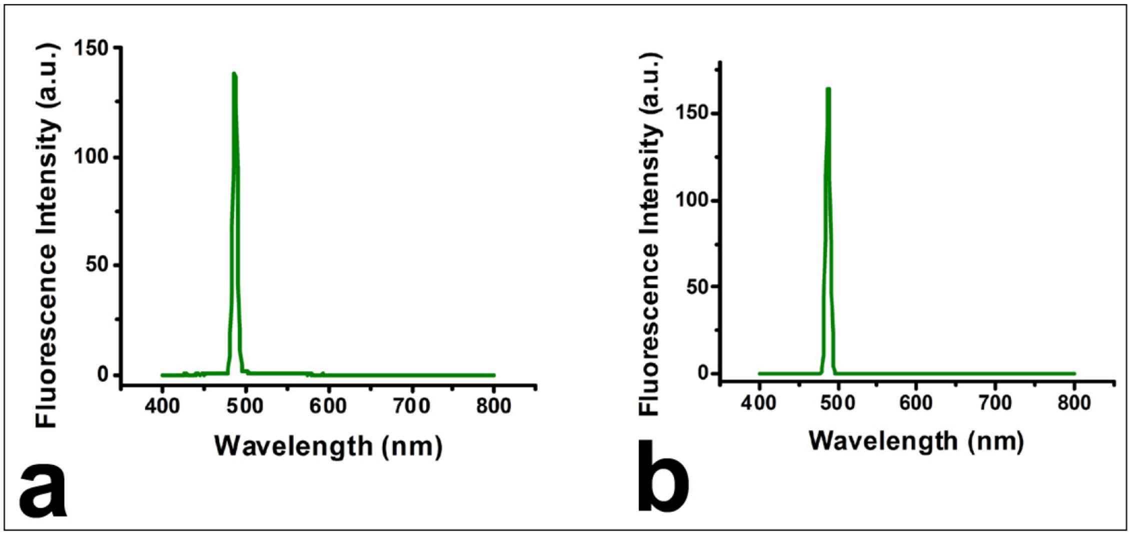

Supplementary Figure S1: The total protein was extracted from the co-transfected (combination of TET1-dCas9 plasmids and sgRNAs) cells, and the emission spectra of the fluorescent tags (EGFP) in the fusion proteins were recorded using a fluorimeter. A slight shift in EGFP emission also suggests the possible formation of TET1-dCas9-EGFP fusion proteins namely TDE-I and TDE-II. Representative emission spectra is presented for TDE-I in HeLa **a.** and MCF7 **b.** cell free extracts.

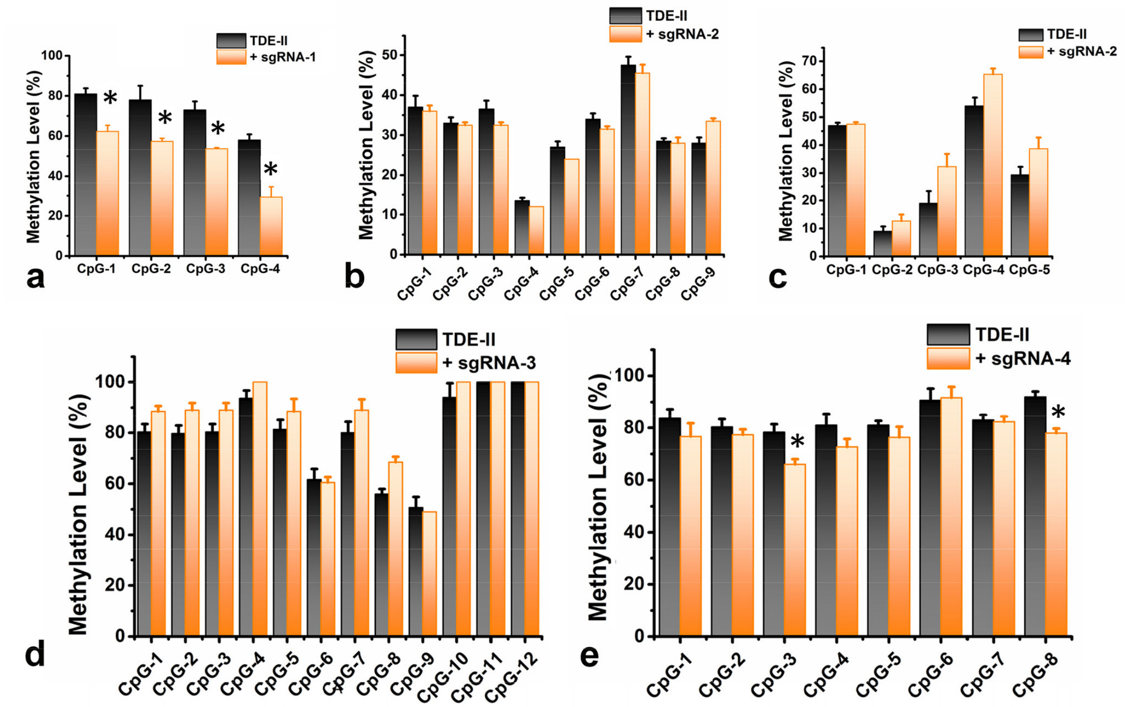

Supplementary Figure S2: Quantitative determination of DNA demethylation levels at the target CpG sites in *BRCA1* promoter. The levels of DNA methylation were determined by pyrosequencing in HeLa cells, treated with TDE-II and different combinations of sgRNAs (in orange), compared to only TDE-II treated ones (in black) (**a-e**). A p value of < 0.05 was considered statistically significant for all the obtained data.

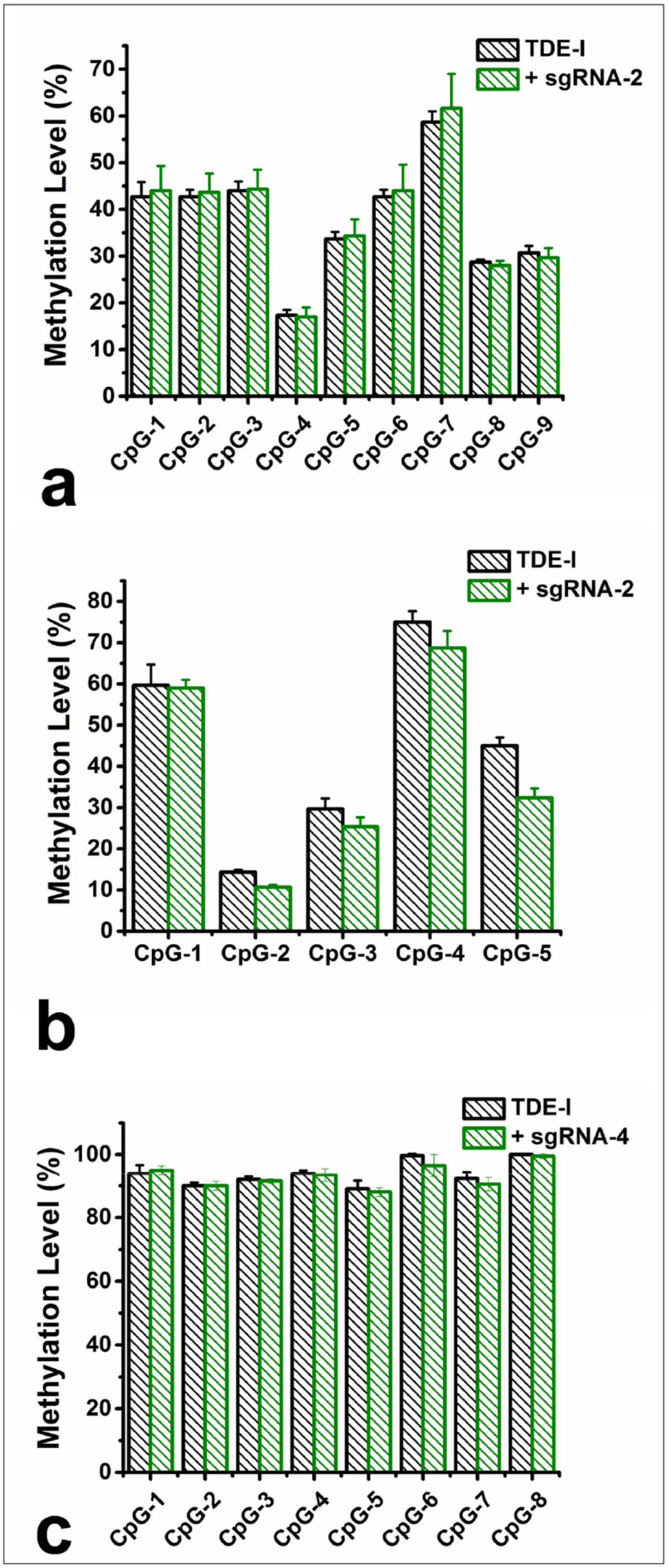

Supplementary Figure S3: Quantitative determination of DNA demethylation level at the target CpG sites in *BRCA1* promoter. The levels of DNA methylation were determined by pyrosequencing in MCF7 cells, treated with the combination of TDE-I and sgRNA-2 (**a, b**), and the combination of TDE-I with sgRNA-4 in MCF-7 cells.

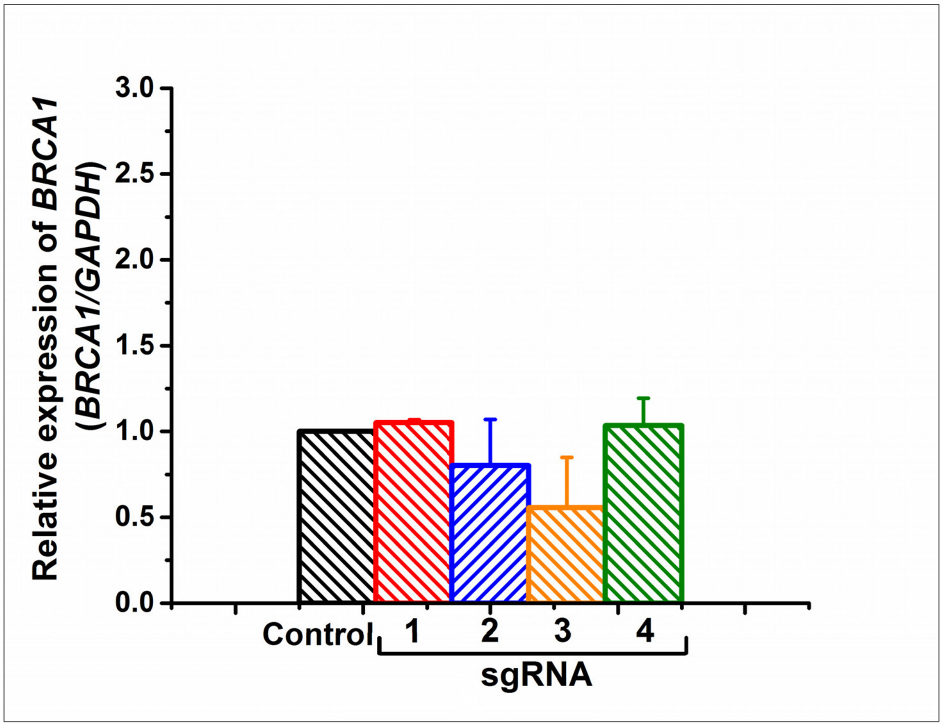

Supplementary Figure S4: The gene expression of *BRCA1* was determined with qPCR analysis followed by the co-treatment with TDE-II and different combinations of sg-RNAs in HeLa cells.

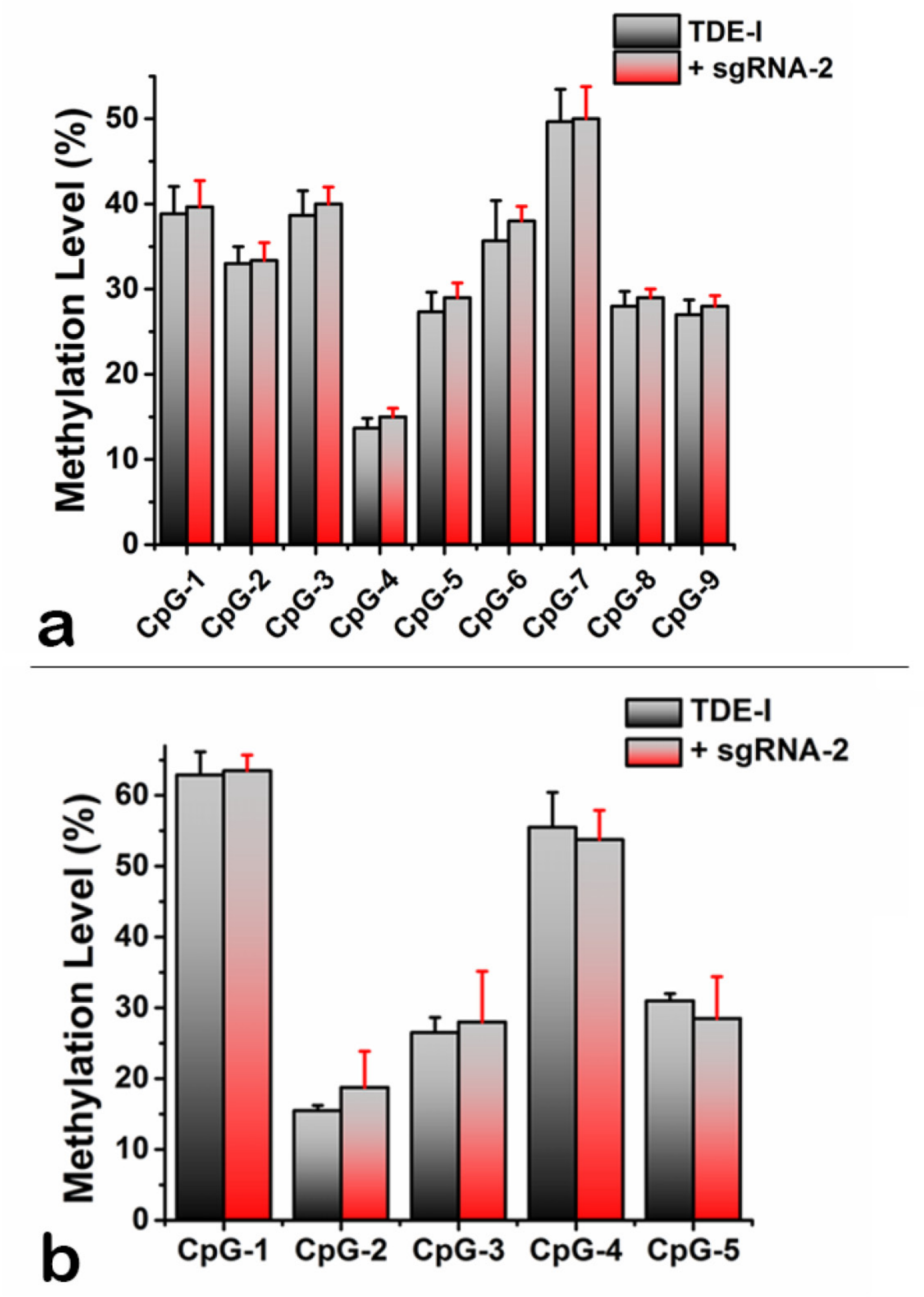

Supplementary Figure S5: Quantitative determination of DNA demethylation level at the target CpG sites in *BRCA1* promoter. The levels of DNA methylation were determined by pyrosequencing in HeLa cells, treated with the combination of inactive TET1 fusion protein and sgRNA-2 (**a**, **b**).

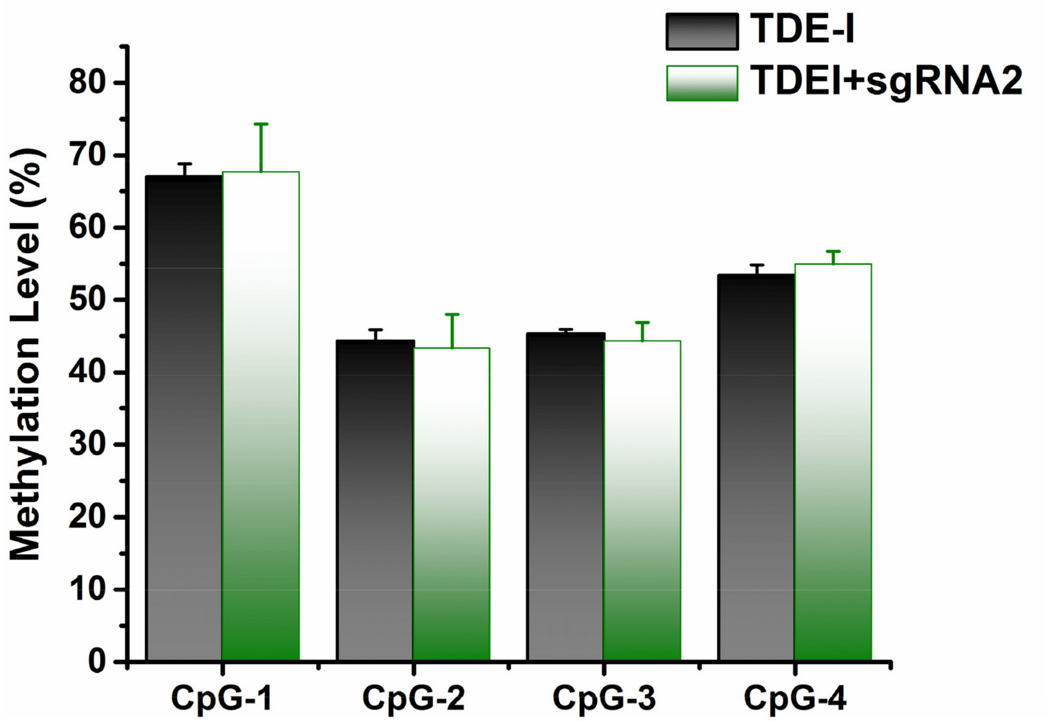

Supplementary Figure S6: Quantitative determination of DNA demethylation levels at the target CpG sites at global LINE-1 repeat sequence element. The levels of DNA methylation were determined by pyrosequencing in HeLa cells, treated with TDE-I and compared to the combination of TDEI + sgRNA-2 (**a-e**). A p value of < 0.05 was considered statistically significant for all of the obtained data

Supplementary Table S1: Primers used in this study for PCR amplification and sequencing analysis of the fusion-protein construct (TET1CD- dCas9-EGFP), all primers listed are 5' to 3'

TET1CD- dCas9-EGFP (TDE) fusion protein construct			
**PCR primers**			
**Insert name**		**Forward**	**Reverse**
**TDE-I**	**TET1CD**	atcgttGGTACCGCCACCATGGAACT GCCCACCTGCAGCTGTCTTGAT	atcgttGGATCCGAC CCAATGGTTAT AGGGCCCCGCAACGTG
	**dCas9**	ATCGTTGGATCC AGCGGAAGTACACCCGCA ATGGCTAGCCCCAAA AAGAAGAGGAAAGTG	atcctaTCTAGA ACCTACCTTGC GCTTTTTCTTGGGAG
**TDE-II**	**TET1CD**	atcgttGGTACC GCCACC ATGGAACTGCC CACCTGCAGCTGTCTTGAT	atcgttGGATCC GACCCAATGGTT ATAGGGCCCCGCAACGTG
	**dCas9**	atcgttGGATCC GCCACC ATGGCTAGCCCC AAAAAGAAGAGGAAAGTG	atcctaTCTAGA ACCTACCTTG CGCTTTTTCTTGGGAG
**Sequencing primers**			
**Primer name**	**Sequence (5' to 3')**	**Primer name**	**Sequence (5' to 3')**
**TDE_SP1**	GAGGGGTTTTATGCGATG	**TDE_SP6**	CGTGAACACTGAA ATCACCAAG
**TDE_SP2**	TCCAAGCTCTCCCTTACATGA	**TDE _SP7**	GTACAACGAGCT GACCAAGGTG
**TDE_SP3**	ACCTTAGGGAGT AACACTGAGACC	**TDE _SP8**	GTATTCTCCAGACCG TGAAAGTC
**TDE_SP4**	ACATTGATGAGTAT TGGTCAGACAG	**TDE _SP9**	CGCGAGGTG AAGGTGATTA
**TDE_SP5**	ATCCCATCTTCGGTAATATCGT	**TDE _SP10**	GAACTGCTGGGT ATCACCATTAT
**Sequence (3' to 5')**			
**TDE_SP11**		TGTACAGCTCGTCCATGC	

The restriction sites are represented in green, linkers in red, and the sites complementary to the inserts in black font.

Supplementary Table S2: Primers used in this study for PCR amplification and sequencing analysis of the fusion-protein dCas9- TET1CD (inactive)-EGFP, all primers listed are 5' to 3'

dCas9- TET1CD (inactive)- EGFP fusion protein construct			
PCR primers			
	**Insert name**	**Forward**	**Reverse**
**dCas9-TET1CD (inactive)-EGFP**	**dCas9**	atcgttCCATGGCTAGCCCC AAAAAGAAGAGGAAAGTG	gctagcGGCGCGCCCACCTA CCTTGCGCTTTTTCTTGGGAG
	**TET1CD (inactive)**	atcgttGCTAGC AGCGGA AGTACACCCGCA ATGGAACTGCCCA CCTGCAGCTGTCTTGAT	atcctaCCTGCAGGGACCCAAT GGTTATAGGGCCCCGCAACGTG
	**EGFP**	atcctaCCTGCAGG AAGCGGAAGTACACCCGCA ATGGTGAGCAAGGGCGAGG	tctacaaaGCGGCCGCCTACT TGTACAGCTCGTCCATG

The restriction sites are represented in green, linkers in red, and the sites complementary to the inserts in black font.

Supplementary Table S3: Primers used in this study for the PCR amplification of *BRCA1* after bisulfite conversion of the HeLa genomic DNA. The target region was amplified in two fragments with 2 sets of primers.

	**BSP-PCR primers**			
	**Forward**	**Reverse**	**Product size (bp)**	**Annealing Temp (Tm)**
Fragment-1 (F1)	**/5BiosG/**GGGTTAGT TAGGGGTGGGGTTA	CCTCTCCCTCCACACTTC	380	58 °C
Fragment-2 (F2)	TATTTTGGTAGTG TTGGAGGAGTT	**/5BiosG/**AACCACCCT AAAACTCACAAAAATTAAA	400	56 °C
	**Pyrosequencing primers**	**Target sequencing CpG sites (highlighted in yellow)**		
**F1-S1**	*CTCTCATCCTATCACTAAAA	CGATTTTTTTCGTG TTTTCGGATAGTTAATCG		
**F1-S2**	*CAATAACCAACT AAAAAACTCCTC	CGAGGTGATAACGTGTTAGT AGTTTTCGTTCGTTTTCGGCGTTT TTTCGGTTTTGGCGTTTATTTTGGTCG		
**F2-S1**	GTGTTGGAGGAGTTT	CGTTATTGCGTTGTGGGGGTTTTT TTTTGGGTTGGTCGAAGTTAGAG TCGGTTTTTTTTGTTTGCG		
**F2-S2**	TGTGGGAATTGGGGT	CGCGTAGCGTTCGTTAGTTAGCGCGAGTT TTAGGTGGGCGCGGGTTTAGCGGGT TTCGTATTTTCGGTTTCG		
**F2-S3**	GTTTAGGGTAGTTAGGGG	CGGGTTAGTAGTTGTAGAGGG TGCGTCGGGTTTTTTAGTATTG TCGGTTCGTTTGTATTTCGTTTGA ATTTTTATCGGGTTTTAGTCG		

Pyrosequencing was then carried out to quantify the methylation percentage at each CpG sites of the analyzed regions. All the primers listed are 5' to 3'. Bisulfite-PCR (BSP) primers were 5'-biotinylated in either forward or reverse primer.

*Sequencing primer binds to the reverse strand. The sequence of corresponding CpG sites of these sequencing primers hence can be read in the reverse direction of the actual sequence.

Supplementary Table S4: Primers used to study transcript quantification of *BRCA1* relative to the endogenous control GAPDH by qPCR

	Forward	Reverse
BRCA1	CAAGGAACCAGGGATGAAATCAG	ATGGCTCCACATGCAAGTTTG
GAPDH	CAGCCTCAAGATCATCAGCA	TGTGGTCATGAGTCCTTCCA

All the primers listed are 5' to 3'.

Supplementary Table S5: Primers used for qPCR to determine locus specific 5-hydroxymethylation (5-hmC) at the target site of *BRCA1* promoter

Treated with TDE-I plus sgRNA-2		
	**Primer sequence**	**Covered 5-hmC sites† (Amplicon size: 270 bp)**
**Forward**	GAGCCCTTCGTGTTCTGAGG	**GCGC**CGGAGAGTTGGAGAGTCTGTGGT TCAGAATGCGAGGTGACAACGTGCTAGC AGCCCTCGCTCGCTCTCG**GCGC**CTCCTCGGCCTT GGCGTCCATTCTGGCCGTGCTGGAGGA GCCCTTCAGCCCGCCACT**GCGC**
**Reverse**	GGTGTGGGAACTGGGGCT	

All the primers listed are 5' to 3'.
