# Supplementary material for: CRISPR-dCas9 mediated TET1 targeting for selective DNA demethylation at BRCA1 promoter
Source: Oncotarget. 2016 Jun 23;7(29):46545–56. doi: 10.18632/oncotarget.10234 (PMC5216816; doi:10.18632/oncotarget.10234)
Supplement: Supplementary file 2 [file oncotarget-07-46545-s002.doc]

**Supporting Information**

**Supplementary sequence-1:** Nucleotide sequences of the **EF1α-TET1CD-dCas9-NLS-2A-EGFP** fusion protein. Restriction sites are highlighted in yellow.

AAGCTTTGCAAAGATGGATAAAGTTTTAAACAGAGAGGAATCTTTGCAGCTAATGGACCTTCTAGGTCTTGAAAGGAGTGGGAATTGGCTCCGGTGCCCGTCAGTGGGCAGAGCGCACATCGCCCACAGTCCCCGAGAAGTTGGGGGGAGGGGTCGGCAATTGAACCGGTGCCTAGAGAAGGTGGCGCGGGGTAAACTGGGAAAGTGATGTCGTGTACTGGCTCCGCCTTTTTCCCGAGGGTGGGGGAGAACCGTATATAAGTGCAGTAGTCGCCGTGAACGTTCTTTTTCGCAACGGGTTTGCCGCCAGAACACAGGTAAGTGCCGTGTGTGGTTCCCGCGGGCCTGGCCTCTTTACGGGTTATGGCCCTTGCGTGCCTTGAATTACTTCCACCTGGCTGCAGTACGTGATTCTTGATCCCGAGCTTCGGGTTGGAAGTGGGTGGGAGAGTTCGAGGCCTTGCGCTTAAGGAGCCCCTTCGCCTCGTGCTTGAGTTGAGGCCTGGCCTGGGCGCTGGGGCCGCCGCGTGCGAATCTGGTGGCACCTTCGCGCCTGTCTCGCTGCTTTCGATAAGTCTCTAGCCATTTAAAATTTTTGATGACCTGCTGCGACGCTTTTTTTCTGGCAAGATAGTCTTGTAAATGCGGGCCAAGATCTGCACACTGGTATTTCGGTTTTTGGGGCCGCGGGCGGCGACGGGGCCCGTGCGTCCCAGCGCACATGTTCGGCGAGGCGGGGCCTGCGAGCGCGGCCACCGAGAATCGGACGGGGGTAGTCTCAAGCTGGCCGGCCTGCTCTGGTGCCTGGCCTCGCGCCGCCGTGTATCGCCCCGCCCTGGGCGGCAAGGCTGGCCCGGTCGGCACCAGTTGCGTGAGCGGAAAGATGGCCGCTTCCCGGCCCTGCTGCAGGGAGCTCAAAATGGAGGACGCGGCGCTCGGGAGAGCGGGCGGGTGAGTCACCCACACAAAGGAAAAGGGCCTTTCCGTCCTCAGCCGTCGCTTCATGTGACTCCACGGAGTACCGGGCGCCGTCCAGGCACCTCGATTAGTTCTCGAGCTTTTGGAGTACGTCGTCTTTAGGTTGGGGGGAGGGGTTTTATGCGATGGAGTTTCCCCACACTGAGTGGGTGGAGACTGAAGTTAGGCCAGCTTGGCACTTGATGTAATTCTCCTTGGAATTTGCCCTTTTTGAGTTTGGATCTTGGTTCATTCTCAAGCCTCAGACAGTGGTTCAAAGTTTTTTTCTTCCATTTCAGGTGTCGTGAGGTACCGCCACCATGGAACTGCCCACCTGCAGCTGTCTTGATCGAGTTATACAAAAAGACAAAGGCCCATATTATACACACCTTGGGGCAGGACCAAGTGTTGCTGCTGTCAGGGAAATCATGGAGAATAGGTATGGTCAAAAAGGAAACGCAATAAGGATAGAAATAGTAGTGTACACCGGTAAAGAAGGGAAAAGCTCTCATGGGTGTCCAATTGCTAAGTGGGTTTTAAGAAGAAGCAGTGATGAAGAAAAAGTTCTTTGTTTGGTCCGGCAGCGTACAGGCCACCACTGTCCAACTGCTGTGATGGTGGTGCTCATCATGGTGTGGGATGGCATCCCTCTTCCAATGGCCGACCGGCTATACACAGAGCTCACAGAGAATCTAAAGTCATACAATGGGCACCCTACCGACAGAAGATGCACCCTCAATGAAAATCGTACCTGTACATGTCAAGGAATTGATCCAGAGACTTGTGGAGCTTCATTCTCTTTTGGCTGTTCATGGAGTATGTACTTTAATGGCTGTAAGTTTGGTAGAAGCCCAAGCCCCAGAAGATTTAGAATTGATCCAAGCTCTCCCTTACATGAAAAAAACCTTGAAGATAACTTACAGAGTTTGGCTACACGATTAGCTCCAATTTATAAGCAGTATGCTCCAGTAGCTTACCAAAATCAGGTGGAATATGAAAATGTTGCCCGAGAATGTCGGCTTGGCAGCAAGGAAGGTCGTCCCTTCTCTGGGGTCACTGCTTGCCTGGACTTCTGTGCTCATCCCCACAGGGACATTCACAACATGAATAATGGAAGCACTGTGGTTTGTACCTTAACTCGAGAAGATAACCGCTCTTTGGGTGTTATTCCTCAAGATGAGCAGCTCCATGTGCTACCTCTTTATAAGCTTTCAGACACAGATGAGTTTGGCTCCAAGGAAGGAATGGAAGCCAAGATCAAATCTGGGGCCATCGAGGTCCTGGCACCCCGCCGCAAAAAAAGAACGTGTTTCACTCAGCCTGTTCCCCGTTCTGGAAAGAAGAGGGCTGCGATGATGACAGAGGTTCTTGCACATAAGATAAGGGCAGTGGAAAAGAAACCTATTCCCCGAATCAAGCGGAAGAATAACTCAACAACAACAAACAACAGTAAGCCTTCGTCACTGCCAACCTTAGGGAGTAACACTGAGACCGTGCAACCTGAAGTAAAAAGTGAAACCGAACCCCATTTTATCTTAAAAAGTTCAGACAACACTAAAACTTATTCGCTGATGCCATCCGCTCCTCACCCAGTGAAAGAGGCATCTCCAGGCTTCTCCTGGTCCCCGAAGACTGCTTCAGCCACACCAGCTCCACTGAAGAATGACGCAACAGCCTCATGCGGGTTTTCAGAAAGAAGCAGCACTCCCCACTGTACGATGCCTTCGGGAAGACTCAGTGGTGCCAATGCAGCTGCTGCTGATGGCCCTGGCATTTCACAGCTTGGCGAAGTGGCTCCTCTCCCCACCCTGTCTGCTCCTGTGATGGAGCCCCTCATTAATTCTGAGCCTTCCACTGGTGTGACTGAGCCGCTAACGCCTCATCAGCCAAACCACCAGCCCTCCTTCCTCACCTCTCCTCAAGACCTTGCCTCTTCTCCAATGGAAGAAGATGAGCAGCATTCTGAAGCAGATGAGCCTCCATCAGACGAACCCCTATCTGATGACCCCCTGTCACCTGCTGAGGAGAAATTGCCCCACATTGATGAGTATTGGTCAGACAGTGAGCACATCTTTTTGGATGCAAATATTGGTGGGGTGGCCATCGCACCTGCTCACGGCTCGGTTTTGATTGAGTGTGCCCGGCGAGAGCTGCACGCTACCACTCCTGTTGAGCACCCCAACCGTAATCATCCAACCCGCCTCTCCCTTGTCTTTTACCAGCACAAAAACCTAAATAAGCCCCAACATGGTTTTGAACTAAACAAGATTAAGTTTGAGGCTAAAGAAGCTAAGAATAAGAAAATGAAGGCCTCAGAGCAAAAAGACCAGGCAGCTAATGAAGGTCCAGAACAGTCCTCTGAAGTAAATGAATTGAACCAAATTCCTTCTCATAAAGCATTAACATTAACCCATGACAATGTTGTCACCGTGTCCCCTTATGCTCTCACACACGTTGCGGGGCCCTATAACCATTGGGTCGGATCC**GCCACCATG**GCTAGCCCCAAAAAGAAGAGGAAAGTGGACAAGAAGTATTCTATCGGACTGGCCATCGGGACTAATAGCGTCGGGTGGGCCGTGATCACTGACGAGTACAAGGTGCCCTCTAAGAAGTTCAAGGTGCTCGGGAACACCGACCGGCATTCCATCAAGAAAAATCTGATCGGAGCTCTCCTCTTTGATTCAGGGGAGACCGCTGAAGCAACCCGCCTCAAGCGGACTGCTAGACGGCGGTACACCAGGAGGAAGAACCGGATTTGTTACCTTCAAGAGATATTCTCCAACGAAATGGCAAAGGTCGACGACAGCTTCTTCCATAGGCTGGAAGAATCATTCCTCGTGGAAGAGGATAAGAAGCATGAACGGCATCCCATCTTCGGTAATATCGTCGACGAGGTGGCCTATCACGAGAAATACCCAACCATCTACCATCTTCGCAAAAAGCTGGTGGACTCAACCGACAAGGCAGACCTCCGGCTTATCTACCTGGCCCTGGCCCACATGATCAAGTTCAGAGGCCACTTCCTGATCGAGGGCGACCTCAATCCTGACAATAGCGATGTGGATAAACTGTTCATCCAGCTGGTGCAGACTTACAACCAGCTCTTTGAAGAGAACCCCATCAATGCAAGCGGAGTCGATGCCAAGGCCATTCTGTCAGCCCGGCTGTCAAAGAGCCGCAGACTTGAGAATCTTATCGCTCAGCTGCCGGGTGAAAAGAAAAATGGACTGTTCGGGAACCTGATTGCTCTTTCACTTGGGCTGACTCCCAATTTCAAGTCTAATTTCGACCTGGCAGAGGATGCCAAGCTGCAACTGTCCAAGGACACCTATGATGACGATCTCGACAACCTCCTGGCCCAGATCGGTGACCAATACGCCGACCTTTTCCTTGCTGCTAAGAATCTTTCTGACGCCATCCTGCTGTCTGACATTCTCCGCGTGAACACTGAAATCACCAAGGCCCCTCTTTCAGCTTCAATGATTAAGCGGTATGATGAGCACCACCAGGACCTGACCCTGCTTAAGGCACTCGTCCGGCAGCAGCTTCCGGAGAAGTACAAGGAAATCTTCTTTGACCAGTCAAAGAATGGATACGCCGGCTACATCGACGGAGGTGCCTCCCAAGAGGAATTTTATAAGTTTATCAAACCTATCCTTGAGAAGATGGACGGCACCGAAGAGCTCCTCGTGAAACTGAATCGGGAGGATCTGCTGCGGAAGCAGCGCACTTTCGACAATGGGAGCATTCCCCACCAGATCCATCTTGGGGAGCTTCACGCCATCCTTCGGCGCCAAGAGGACTTCTACCCCTTTCTTAAGGACAACAGGGAGAAGATTGAGAAAATTCTCACTTTCCGCATCCCCTACTACGTGGGACCCCTCGCCAGAGGAAATAGCCGGTTTGCTTGGATGACCAGAAAGTCAGAAGAAACTATCACTCCCTGGAACTTCGAAGAGGTGGTGGACAAGGGAGCCAGCGCTCAGTCATTCATCGAACGGATGACTAACTTCGATAAGAACCTCCCCAATGAGAAGGTCCTGCCGAAACATTCCCTGCTCTACGAGTACTTTACCGTGTACAACGAGCTGACCAAGGTGAAATATGTCACCGAAGGGATGAGGAAGCCCGCATTCCTGTCAGGCGAACAAAAGAAGGCAATTGTGGACCTTCTGTTCAAGACCAATAGAAAGGTGACCGTGAAGCAGCTGAAGGAGGACTATTTCAAGAAAATTGAATGCTTCGACTCTGTGGAGATTAGCGGGGTCGAAGATCGGTTCAACGCAAGCCTGGGTACCTACCATGATCTGCTTAAGATCATCAAGGACAAGGATTTTCTGGACAATGAGGAGAACGAGGACATCCTTGAGGACATTGTCCTGACTCTCACTCTGTTCGAGGACCGGGAAATGATCGAGGAGAGGCTTAAGACCTACGCCCATCTGTTCGACGATAAAGTGATGAAGCAACTTAAACGGAGAAGATATACCGGATGGGGACGCCTTAGCCGCAAACTCATCAACGGAATCCGGGACAAACAGAGCGGAAAGACCATTCTTGATTTCCTTAAGAGCGACGGATTCGCTAATCGCAACTTCATGCAACTTATCCATGATGATTCCCTGACCTTTAAGGAGGACATCCAGAAGGCCCAAGTGTCTGGACAAGGTGACTCACTGCACGAGCATATCGCAAATCTGGCTGGTTCACCCGCTATTAAGAAGGGTATTCTCCAGACCGTGAAAGTCGTGGACGAGCTGGTCAAGGTGATGGGTCGCCATAAACCAGAGAACATTGTCATCGAGATGGCCAGGGAAAACCAGACTACCCAGAAGGGACAGAAGAACAGCAGGGAGCGGATGAAAAGAATTGAGGAAGGGATTAAGGAGCTCGGGTCACAGATCCTTAAAGAGCACCCGGTGGAAAACACCCAGCTTCAGAATGAGAAGCTCTATCTGTACTACCTTCAAAATGGACGCGATATGTATGTGGACCAAGAGCTTGATATCAACAGGCTCTCAGACTACGACGTGGACGCCATCGTCCCTCAGAGCTTCCTCAAAGACGACTCAATTGACAATAAGGTGCTGACTCGCTCAGACAAGAACCGGGGAAAGTCAGATAACGTGCCCTCAGAGGAAGTCGTGAAAAAGATGAAGAACTATTGGCGCCAGCTTCTGAACGCAAAGCTGATCACTCAGCGGAAGTTCGACAATCTCACTAAGGCTGAGAGGGGCGGACTGAGCGAACTGGACAAAGCAGGATTCATTAAACGGCAACTTGTGGAGACTCGGCAGATTACTAAACATGTCGCCCAAATCCTTGACTCACGCATGAATACCAAGTACGACGAAAACGACAAACTTATCCGCGAGGTGAAGGTGATTACCCTGAAGTCCAAGCTGGTCAGCGATTTCAGAAAGGACTTTCAATTCTACAAAGTGCGGGAGATCAATAACTATCATCATGCTCATGACGCATATCTGAATGCCGTGGTGGGAACCGCCCTGATCAAGAAGTACCCAAAGCTGGAAAGCGAGTTCGTGTACGGAGACTACAAGGTCTACGACGTGCGCAAGATGATTGCCAAATCTGAGCAGGAGATCGGAAAGGCCACCGCAAAGTACTTCTTCTACAGCAACATCATGAATTTCTTCAAGACCGAAATCACCCTTGCAAACGGTGAGATCCGGAAGAGGCCGCTCATCGAGACTAATGGGGAGACTGGCGAAATCGTGTGGGACAAGGGCAGAGATTTCGCTACCGTGCGCAAAGTGCTTTCTATGCCTCAAGTGAACATCGTGAAGAAAACCGAGGTGCAAACCGGAGGCTTTTCTAAGGAATCAATCCTCCCCAAGCGCAACTCCGACAAGCTCATTGCAAGGAAGAAGGATTGGGACCCTAAGAAGTACGGCGGATTCGATTCACCAACTGTGGCTTATTCTGTCCTGGTCGTGGCTAAGGTGGAAAAAGGAAAGTCTAAGAAGCTCAAGAGCGTGAAGGAACTGCTGGGTATCACCATTATGGAGCGCAGCTCCTTCGAGAAGAACCCAATTGACTTTCTCGAAGCCAAAGGTTACAAGGAAGTCAAGAAGGACCTTATCATCAAGCTCCCAAAGTATAGCCTGTTCGAACTGGAGAATGGGCGGAAGCGGATGCTCGCCTCCGCTGGCGAACTTCAGAAGGGTAATGAGCTGGCTCTCCCCTCCAAGTACGTGAATTTCCTCTACCTTGCAAGCCATTACGAGAAGCTGAAGGGGAGCCCCGAGGACAACGAGCAAAAGCAACTGTTTGTGGAGCAGCATAAGCATTATCTGGACGAGATCATTGAGCAGATTTCCGAGTTTTCTAAACGCGTCATTCTCGCTGATGCCAACCTCGATAAAGTCCTTAGCGCATACAATAAGCACAGAGACAAACCAATTCGGGAGCAGGCTGAGAATATCATCCACCTGTTCACCCTCACCAATCTTGGTGCCCCTGCCGCATTCAAGTACTTCGACACCACCATCGACCGGAAACGCTATACCTCCACCAAAGAAGTGCTGGACGCCACCCTCATCCACCAGAGCATCACCGGACTTTACGAAACTCGGATTGACCTCTCACAGCTCGGAGGGGATGAGGGAGCT**CCCAAGAAAAAGCGCAAGGT**AGGTTCTAGAGGCAGTGGAGAGGGCAGAGGAAGTCTGCTAACATGCGGTGACGTCGAGGAGAATCCTGGCCCAGTGAGCAAGGGCGAGGAGCTGTTCACCGGGGTGGTGCCCATCCTGGTCGAGCTGGACGGCGACGTAAACGGCCACAAGTTCAGCGTGTCCGGCGAGGGCGAGGGCGATGCCACCTACGGCAAGCTGACCCTGAAGTTCATCTGCACCACCGGCAAGCTGCCCGTGCCCTGGCCCACCCTCGTGACCACCCTGACCTACGGCGTGCAGTGCTTCAGCCGCTACCCCGACCACATGAAGCAGCACGACTTCTTCAAGTCCGCCATGCCCGAAGGCTACGTCCAGGAGCGCACCATCTTCTTCAAGGACGACGGCAACTACAAGACCCGCGCCGAGGTGAAGTTCGAGGGCGACACCCTGGTGAACCGCATCGAGCTGAAGGGCATCGACTTCAAGGAGGACGGCAACATCCTGGGGCACAAGCTGGAGTACAACTACAACAGCCACAACGTCTATATCATGGCCGACAAGCAGAAGAACGGCATCAAGGTGAACTTCAAGATCCGCCACAACATCGAGGACGGCAGCGTGCAGCTCGCCGACCACTACCAGCAGAACACCCCCATCGGCGACGGCCCCGTGCTGCTGCCCGACAACCACTACCTGAGCACCCAGTCCGCCCTGAGCAAAGACCCCAACGAGAAGCGCGATCACATGGTCCTGCTGGAGTTCGTGACCGCCGCCGGGATCACTCTCGGCATGGACGAGCTGTACAagtaaGAATTC

**Supplementary sequence-2:** Nucleotide sequences of the **EF1α-TET1CD-linker- dCas9-NLS-2A-EGFP** fusion protein. Restriction sites are highlighted in yellow.

AAGCTTTGCAAAGATGGATAAAGTTTTAAACAGAGAGGAATCTTTGCAGCTAATGGACCTTCTAGGTCTTGAAAGGAGTGGGAATTGGCTCCGGTGCCCGTCAGTGGGCAGAGCGCACATCGCCCACAGTCCCCGAGAAGTTGGGGGGAGGGGTCGGCAATTGAACCGGTGCCTAGAGAAGGTGGCGCGGGGTAAACTGGGAAAGTGATGTCGTGTACTGGCTCCGCCTTTTTCCCGAGGGTGGGGGAGAACCGTATATAAGTGCAGTAGTCGCCGTGAACGTTCTTTTTCGCAACGGGTTTGCCGCCAGAACACAGGTAAGTGCCGTGTGTGGTTCCCGCGGGCCTGGCCTCTTTACGGGTTATGGCCCTTGCGTGCCTTGAATTACTTCCACCTGGCTGCAGTACGTGATTCTTGATCCCGAGCTTCGGGTTGGAAGTGGGTGGGAGAGTTCGAGGCCTTGCGCTTAAGGAGCCCCTTCGCCTCGTGCTTGAGTTGAGGCCTGGCCTGGGCGCTGGGGCCGCCGCGTGCGAATCTGGTGGCACCTTCGCGCCTGTCTCGCTGCTTTCGATAAGTCTCTAGCCATTTAAAATTTTTGATGACCTGCTGCGACGCTTTTTTTCTGGCAAGATAGTCTTGTAAATGCGGGCCAAGATCTGCACACTGGTATTTCGGTTTTTGGGGCCGCGGGCGGCGACGGGGCCCGTGCGTCCCAGCGCACATGTTCGGCGAGGCGGGGCCTGCGAGCGCGGCCACCGAGAATCGGACGGGGGTAGTCTCAAGCTGGCCGGCCTGCTCTGGTGCCTGGCCTCGCGCCGCCGTGTATCGCCCCGCCCTGGGCGGCAAGGCTGGCCCGGTCGGCACCAGTTGCGTGAGCGGAAAGATGGCCGCTTCCCGGCCCTGCTGCAGGGAGCTCAAAATGGAGGACGCGGCGCTCGGGAGAGCGGGCGGGTGAGTCACCCACACAAAGGAAAAGGGCCTTTCCGTCCTCAGCCGTCGCTTCATGTGACTCCACGGAGTACCGGGCGCCGTCCAGGCACCTCGATTAGTTCTCGAGCTTTTGGAGTACGTCGTCTTTAGGTTGGGGGGAGGGGTTTTATGCGATGGAGTTTCCCCACACTGAGTGGGTGGAGACTGAAGTTAGGCCAGCTTGGCACTTGATGTAATTCTCCTTGGAATTTGCCCTTTTTGAGTTTGGATCTTGGTTCATTCTCAAGCCTCAGACAGTGGTTCAAAGTTTTTTTCTTCCATTTCAGGTGTCGTGAGGTACCGCCACCATGGAACTGCCCACCTGCAGCTGTCTTGATCGAGTTATACAAAAAGACAAAGGCCCATATTATACACACCTTGGGGCAGGACCAAGTGTTGCTGCTGTCAGGGAAATCATGGAGAATAGGTATGGTCAAAAAGGAAACGCAATAAGGATAGAAATAGTAGTGTACACCGGTAAAGAAGGGAAAAGCTCTCATGGGTGTCCAATTGCTAAGTGGGTTTTAAGAAGAAGCAGTGATGAAGAAAAAGTTCTTTGTTTGGTCCGGCAGCGTACAGGCCACCACTGTCCAACTGCTGTGATGGTGGTGCTCATCATGGTGTGGGATGGCATCCCTCTTCCAATGGCCGACCGGCTATACACAGAGCTCACAGAGAATCTAAAGTCATACAATGGGCACCCTACCGACAGAAGATGCACCCTCAATGAAAATCGTACCTGTACATGTCAAGGAATTGATCCAGAGACTTGTGGAGCTTCATTCTCTTTTGGCTGTTCATGGAGTATGTACTTTAATGGCTGTAAGTTTGGTAGAAGCCCAAGCCCCAGAAGATTTAGAATTGATCCAAGCTCTCCCTTACATGAAAAAAACCTTGAAGATAACTTACAGAGTTTGGCTACACGATTAGCTCCAATTTATAAGCAGTATGCTCCAGTAGCTTACCAAAATCAGGTGGAATATGAAAATGTTGCCCGAGAATGTCGGCTTGGCAGCAAGGAAGGTCGTCCCTTCTCTGGGGTCACTGCTTGCCTGGACTTCTGTGCTCATCCCCACAGGGACATTCACAACATGAATAATGGAAGCACTGTGGTTTGTACCTTAACTCGAGAAGATAACCGCTCTTTGGGTGTTATTCCTCAAGATGAGCAGCTCCATGTGCTACCTCTTTATAAGCTTTCAGACACAGATGAGTTTGGCTCCAAGGAAGGAATGGAAGCCAAGATCAAATCTGGGGCCATCGAGGTCCTGGCACCCCGCCGCAAAAAAAGAACGTGTTTCACTCAGCCTGTTCCCCGTTCTGGAAAGAAGAGGGCTGCGATGATGACAGAGGTTCTTGCACATAAGATAAGGGCAGTGGAAAAGAAACCTATTCCCCGAATCAAGCGGAAGAATAACTCAACAACAACAAACAACAGTAAGCCTTCGTCACTGCCAACCTTAGGGAGTAACACTGAGACCGTGCAACCTGAAGTAAAAAGTGAAACCGAACCCCATTTTATCTTAAAAAGTTCAGACAACACTAAAACTTATTCGCTGATGCCATCCGCTCCTCACCCAGTGAAAGAGGCATCTCCAGGCTTCTCCTGGTCCCCGAAGACTGCTTCAGCCACACCAGCTCCACTGAAGAATGACGCAACAGCCTCATGCGGGTTTTCAGAAAGAAGCAGCACTCCCCACTGTACGATGCCTTCGGGAAGACTCAGTGGTGCCAATGCAGCTGCTGCTGATGGCCCTGGCATTTCACAGCTTGGCGAAGTGGCTCCTCTCCCCACCCTGTCTGCTCCTGTGATGGAGCCCCTCATTAATTCTGAGCCTTCCACTGGTGTGACTGAGCCGCTAACGCCTCATCAGCCAAACCACCAGCCCTCCTTCCTCACCTCTCCTCAAGACCTTGCCTCTTCTCCAATGGAAGAAGATGAGCAGCATTCTGAAGCAGATGAGCCTCCATCAGACGAACCCCTATCTGATGACCCCCTGTCACCTGCTGAGGAGAAATTGCCCCACATTGATGAGTATTGGTCAGACAGTGAGCACATCTTTTTGGATGCAAATATTGGTGGGGTGGCCATCGCACCTGCTCACGGCTCGGTTTTGATTGAGTGTGCCCGGCGAGAGCTGCACGCTACCACTCCTGTTGAGCACCCCAACCGTAATCATCCAACCCGCCTCTCCCTTGTCTTTTACCAGCACAAAAACCTAAATAAGCCCCAACATGGTTTTGAACTAAACAAGATTAAGTTTGAGGCTAAAGAAGCTAAGAATAAGAAAATGAAGGCCTCAGAGCAAAAAGACCAGGCAGCTAATGAAGGTCCAGAACAGTCCTCTGAAGTAAATGAATTGAACCAAATTCCTTCTCATAAAGCATTAACATTAACCCATGACAATGTTGTCACCGTGTCCCCTTATGCTCTCACACACGTTGCGGGGCCCTATAACCATTGGGTCGGATCC**AGCGGAAGTACACCCGCAATG**GCTAGCCCCAAAAAGAAGAGGAAAGTGGACAAGAAGTATTCTATCGGACTGGCCATCGGGACTAATAGCGTCGGGTGGGCCGTGATCACTGACGAGTACAAGGTGCCCTCTAAGAAGTTCAAGGTGCTCGGGAACACCGACCGGCATTCCATCAAGAAAAATCTGATCGGAGCTCTCCTCTTTGATTCAGGGGAGACCGCTGAAGCAACCCGCCTCAAGCGGACTGCTAGACGGCGGTACACCAGGAGGAAGAACCGGATTTGTTACCTTCAAGAGATATTCTCCAACGAAATGGCAAAGGTCGACGACAGCTTCTTCCATAGGCTGGAAGAATCATTCCTCGTGGAAGAGGATAAGAAGCATGAACGGCATCCCATCTTCGGTAATATCGTCGACGAGGTGGCCTATCACGAGAAATACCCAACCATCTACCATCTTCGCAAAAAGCTGGTGGACTCAACCGACAAGGCAGACCTCCGGCTTATCTACCTGGCCCTGGCCCACATGATCAAGTTCAGAGGCCACTTCCTGATCGAGGGCGACCTCAATCCTGACAATAGCGATGTGGATAAACTGTTCATCCAGCTGGTGCAGACTTACAACCAGCTCTTTGAAGAGAACCCCATCAATGCAAGCGGAGTCGATGCCAAGGCCATTCTGTCAGCCCGGCTGTCAAAGAGCCGCAGACTTGAGAATCTTATCGCTCAGCTGCCGGGTGAAAAGAAAAATGGACTGTTCGGGAACCTGATTGCTCTTTCACTTGGGCTGACTCCCAATTTCAAGTCTAATTTCGACCTGGCAGAGGATGCCAAGCTGCAACTGTCCAAGGACACCTATGATGACGATCTCGACAACCTCCTGGCCCAGATCGGTGACCAATACGCCGACCTTTTCCTTGCTGCTAAGAATCTTTCTGACGCCATCCTGCTGTCTGACATTCTCCGCGTGAACACTGAAATCACCAAGGCCCCTCTTTCAGCTTCAATGATTAAGCGGTATGATGAGCACCACCAGGACCTGACCCTGCTTAAGGCACTCGTCCGGCAGCAGCTTCCGGAGAAGTACAAGGAAATCTTCTTTGACCAGTCAAAGAATGGATACGCCGGCTACATCGACGGAGGTGCCTCCCAAGAGGAATTTTATAAGTTTATCAAACCTATCCTTGAGAAGATGGACGGCACCGAAGAGCTCCTCGTGAAACTGAATCGGGAGGATCTGCTGCGGAAGCAGCGCACTTTCGACAATGGGAGCATTCCCCACCAGATCCATCTTGGGGAGCTTCACGCCATCCTTCGGCGCCAAGAGGACTTCTACCCCTTTCTTAAGGACAACAGGGAGAAGATTGAGAAAATTCTCACTTTCCGCATCCCCTACTACGTGGGACCCCTCGCCAGAGGAAATAGCCGGTTTGCTTGGATGACCAGAAAGTCAGAAGAAACTATCACTCCCTGGAACTTCGAAGAGGTGGTGGACAAGGGAGCCAGCGCTCAGTCATTCATCGAACGGATGACTAACTTCGATAAGAACCTCCCCAATGAGAAGGTCCTGCCGAAACATTCCCTGCTCTACGAGTACTTTACCGTGTACAACGAGCTGACCAAGGTGAAATATGTCACCGAAGGGATGAGGAAGCCCGCATTCCTGTCAGGCGAACAAAAGAAGGCAATTGTGGACCTTCTGTTCAAGACCAATAGAAAGGTGACCGTGAAGCAGCTGAAGGAGGACTATTTCAAGAAAATTGAATGCTTCGACTCTGTGGAGATTAGCGGGGTCGAAGATCGGTTCAACGCAAGCCTGGGTACCTACCATGATCTGCTTAAGATCATCAAGGACAAGGATTTTCTGGACAATGAGGAGAACGAGGACATCCTTGAGGACATTGTCCTGACTCTCACTCTGTTCGAGGACCGGGAAATGATCGAGGAGAGGCTTAAGACCTACGCCCATCTGTTCGACGATAAAGTGATGAAGCAACTTAAACGGAGAAGATATACCGGATGGGGACGCCTTAGCCGCAAACTCATCAACGGAATCCGGGACAAACAGAGCGGAAAGACCATTCTTGATTTCCTTAAGAGCGACGGATTCGCTAATCGCAACTTCATGCAACTTATCCATGATGATTCCCTGACCTTTAAGGAGGACATCCAGAAGGCCCAAGTGTCTGGACAAGGTGACTCACTGCACGAGCATATCGCAAATCTGGCTGGTTCACCCGCTATTAAGAAGGGTATTCTCCAGACCGTGAAAGTCGTGGACGAGCTGGTCAAGGTGATGGGTCGCCATAAACCAGAGAACATTGTCATCGAGATGGCCAGGGAAAACCAGACTACCCAGAAGGGACAGAAGAACAGCAGGGAGCGGATGAAAAGAATTGAGGAAGGGATTAAGGAGCTCGGGTCACAGATCCTTAAAGAGCACCCGGTGGAAAACACCCAGCTTCAGAATGAGAAGCTCTATCTGTACTACCTTCAAAATGGACGCGATATGTATGTGGACCAAGAGCTTGATATCAACAGGCTCTCAGACTACGACGTGGACGCCATCGTCCCTCAGAGCTTCCTCAAAGACGACTCAATTGACAATAAGGTGCTGACTCGCTCAGACAAGAACCGGGGAAAGTCAGATAACGTGCCCTCAGAGGAAGTCGTGAAAAAGATGAAGAACTATTGGCGCCAGCTTCTGAACGCAAAGCTGATCACTCAGCGGAAGTTCGACAATCTCACTAAGGCTGAGAGGGGCGGACTGAGCGAACTGGACAAAGCAGGATTCATTAAACGGCAACTTGTGGAGACTCGGCAGATTACTAAACATGTCGCCCAAATCCTTGACTCACGCATGAATACCAAGTACGACGAAAACGACAAACTTATCCGCGAGGTGAAGGTGATTACCCTGAAGTCCAAGCTGGTCAGCGATTTCAGAAAGGACTTTCAATTCTACAAAGTGCGGGAGATCAATAACTATCATCATGCTCATGACGCATATCTGAATGCCGTGGTGGGAACCGCCCTGATCAAGAAGTACCCAAAGCTGGAAAGCGAGTTCGTGTACGGAGACTACAAGGTCTACGACGTGCGCAAGATGATTGCCAAATCTGAGCAGGAGATCGGAAAGGCCACCGCAAAGTACTTCTTCTACAGCAACATCATGAATTTCTTCAAGACCGAAATCACCCTTGCAAACGGTGAGATCCGGAAGAGGCCGCTCATCGAGACTAATGGGGAGACTGGCGAAATCGTGTGGGACAAGGGCAGAGATTTCGCTACCGTGCGCAAAGTGCTTTCTATGCCTCAAGTGAACATCGTGAAGAAAACCGAGGTGCAAACCGGAGGCTTTTCTAAGGAATCAATCCTCCCCAAGCGCAACTCCGACAAGCTCATTGCAAGGAAGAAGGATTGGGACCCTAAGAAGTACGGCGGATTCGATTCACCAACTGTGGCTTATTCTGTCCTGGTCGTGGCTAAGGTGGAAAAAGGAAAGTCTAAGAAGCTCAAGAGCGTGAAGGAACTGCTGGGTATCACCATTATGGAGCGCAGCTCCTTCGAGAAGAACCCAATTGACTTTCTCGAAGCCAAAGGTTACAAGGAAGTCAAGAAGGACCTTATCATCAAGCTCCCAAAGTATAGCCTGTTCGAACTGGAGAATGGGCGGAAGCGGATGCTCGCCTCCGCTGGCGAACTTCAGAAGGGTAATGAGCTGGCTCTCCCCTCCAAGTACGTGAATTTCCTCTACCTTGCAAGCCATTACGAGAAGCTGAAGGGGAGCCCCGAGGACAACGAGCAAAAGCAACTGTTTGTGGAGCAGCATAAGCATTATCTGGACGAGATCATTGAGCAGATTTCCGAGTTTTCTAAACGCGTCATTCTCGCTGATGCCAACCTCGATAAAGTCCTTAGCGCATACAATAAGCACAGAGACAAACCAATTCGGGAGCAGGCTGAGAATATCATCCACCTGTTCACCCTCACCAATCTTGGTGCCCCTGCCGCATTCAAGTACTTCGACACCACCATCGACCGGAAACGCTATACCTCCACCAAAGAAGTGCTGGACGCCACCCTCATCCACCAGAGCATCACCGGACTTTACGAAACTCGGATTGACCTCTCACAGCTCGGAGGGGATGAGGGAGCT**CCCAAGAAAAAGCGCAAGGT**AGGTTCTAGAGGCAGTGGAGAGGGCAGAGGAAGTCTGCTAACATGCGGTGACGTCGAGGAGAATCCTGGCCCAGTGAGCAAGGGCGAGGAGCTGTTCACCGGGGTGGTGCCCATCCTGGTCGAGCTGGACGGCGACGTAAACGGCCACAAGTTCAGCGTGTCCGGCGAGGGCGAGGGCGATGCCACCTACGGCAAGCTGACCCTGAAGTTCATCTGCACCACCGGCAAGCTGCCCGTGCCCTGGCCCACCCTCGTGACCACCCTGACCTACGGCGTGCAGTGCTTCAGCCGCTACCCCGACCACATGAAGCAGCACGACTTCTTCAAGTCCGCCATGCCCGAAGGCTACGTCCAGGAGCGCACCATCTTCTTCAAGGACGACGGCAACTACAAGACCCGCGCCGAGGTGAAGTTCGAGGGCGACACCCTGGTGAACCGCATCGAGCTGAAGGGCATCGACTTCAAGGAGGACGGCAACATCCTGGGGCACAAGCTGGAGTACAACTACAACAGCCACAACGTCTATATCATGGCCGACAAGCAGAAGAACGGCATCAAGGTGAACTTCAAGATCCGCCACAACATCGAGGACGGCAGCGTGCAGCTCGCCGACCACTACCAGCAGAACACCCCCATCGGCGACGGCCCCGTGCTGCTGCCCGACAACCACTACCTGAGCACCCAGTCCGCCCTGAGCAAAGACCCCAACGAGAAGCGCGATCACATGGTCCTGCTGGAGTTCGTGACCGCCGCCGGGATCACTCTCGGCATGGACGAGCTGTACAagtaaGAATTC

**Supplementary sequence-3:** Nucleotide sequences of the **CMV-dCas9- TET1CD_inactive_-EGFP-3X.NLS**-**Myc epitope**-**STOP** fusion protein. Restriction sites are highlighted in yellow, and linker sequences are highlighted in green.

GTACCGAATTCACATTGATTATTGAGTAGTTATTAATAGTAATCAATTACGGGGTCATTAGTTCATAGCCCATATATGGAGTTCCGCGTTACATAACTTACGGTAAATGGCCCGCCTGGCTGACCGCCCAACGACCCCCGCCCATTGACGTCAATAATGACGTATGTTCCCATAGTAACGCCAATAGGGACTTTCCATTGACGTCAATGGGTGGAGTATTTACGGTAAACTGCCCACTTGGCAGTACATCAAGTGTATCATATGCCAAGTACGCCCCCTATTGACGTCAATGACGGTAAATGGCCCGCCTGGCATTATGCCCAGTACATGACCTTATGGGACTTTCCTACTTGGCAGTACATCTACGGTTAGTCATCGCTATTACCATAGTGATGCGGTTTTGGCAGTACATCAATGGGCGTGGATAGCGGTTTGACTCACGGGGATTTCCAAGTCTCCACCCCATTGACGTCAATGGGAGTTTGTTTTGGCACCAAAATCAACGGGACTTTCCAAAATGTCGTAACAACTCCGCCCCATTGACGCAAATGGGCGGTAGGCGTGTACGGTGGGAGGTCTATATAAGCAGAGCTTTCTGGCTAACTAGAGAACCCACTGCTTACTGGCACGTGGAAATTAATACGACGTGGCCACCATGGCTAGCCCCAAAAAGAAGAGGAAAGTGGACAAGAAGTATTCTATCGGACTGGCCATCGGGACTAATAGCGTCGGGTGGGCCGTGATCACTGACGAGTACAAGGTGCCCTCTAAGAAGTTCAAGGTGCTCGGGAACACCGACCGGCATTCCATCAAGAAAAATCTGATCGGAGCTCTCCTCTTTGATTCAGGGGAGACCGCTGAAGCAACCCGCCTCAAGCGGACTGCTAGACGGCGGTACACCAGGAGGAAGAACCGGATTTGTTACCTTCAAGAGATATTCTCCAACGAAATGGCAAAGGTCGACGACAGCTTCTTCCATAGGCTGGAAGAATCATTCCTCGTGGAAGAGGATAAGAAGCATGAACGGCATCCCATCTTCGGTAATATCGTCGACGAGGTGGCCTATCACGAGAAATACCCAACCATCTACCATCTTCGCAAAAAGCTGGTGGACTCAACCGACAAGGCAGACCTCCGGCTTATCTACCTGGCCCTGGCCCACATGATCAAGTTCAGAGGCCACTTCCTGATCGAGGGCGACCTCAATCCTGACAATAGCGATGTGGATAAACTGTTCATCCAGCTGGTGCAGACTTACAACCAGCTCTTTGAAGAGAACCCCATCAATGCAAGCGGAGTCGATGCCAAGGCCATTCTGTCAGCCCGGCTGTCAAAGAGCCGCAGACTTGAGAATCTTATCGCTCAGCTGCCGGGTGAAAAGAAAAATGGACTGTTCGGGAACCTGATTGCTCTTTCACTTGGGCTGACTCCCAATTTCAAGTCTAATTTCGACCTGGCAGAGGATGCCAAGCTGCAACTGTCCAAGGACACCTATGATGACGATCTCGACAACCTCCTGGCCCAGATCGGTGACCAATACGCCGACCTTTTCCTTGCTGCTAAGAATCTTTCTGACGCCATCCTGCTGTCTGACATTCTCCGCGTGAACACTGAAATCACCAAGGCCCCTCTTTCAGCTTCAATGATTAAGCGGTATGATGAGCACCACCAGGACCTGACCCTGCTTAAGGCACTCGTCCGGCAGCAGCTTCCGGAGAAGTACAAGGAAATCTTCTTTGACCAGTCAAAGAATGGATACGCCGGCTACATCGACGGAGGTGCCTCCCAAGAGGAATTTTATAAGTTTATCAAACCTATCCTTGAGAAGATGGACGGCACCGAAGAGCTCCTCGTGAAACTGAATCGGGAGGATCTGCTGCGGAAGCAGCGCACTTTCGACAATGGGAGCATTCCCCACCAGATCCATCTTGGGGAGCTTCACGCCATCCTTCGGCGCCAAGAGGACTTCTACCCCTTTCTTAAGGACAACAGGGAGAAGATTGAGAAAATTCTCACTTTCCGCATCCCCTACTACGTGGGACCCCTCGCCAGAGGAAATAGCCGGTTTGCTTGGATGACCAGAAAGTCAGAAGAAACTATCACTCCCTGGAACTTCGAAGAGGTGGTGGACAAGGGAGCCAGCGCTCAGTCATTCATCGAACGGATGACTAACTTCGATAAGAACCTCCCCAATGAGAAGGTCCTGCCGAAACATTCCCTGCTCTACGAGTACTTTACCGTGTACAACGAGCTGACCAAGGTGAAATATGTCACCGAAGGGATGAGGAAGCCCGCATTCCTGTCAGGCGAACAAAAGAAGGCAATTGTGGACCTTCTGTTCAAGACCAATAGAAAGGTGACCGTGAAGCAGCTGAAGGAGGACTATTTCAAGAAAATTGAATGCTTCGACTCTGTGGAGATTAGCGGGGTCGAAGATCGGTTCAACGCAAGCCTGGGTACCTACCATGATCTGCTTAAGATCATCAAGGACAAGGATTTTCTGGACAATGAGGAGAACGAGGACATCCTTGAGGACATTGTCCTGACTCTCACTCTGTTCGAGGACCGGGAAATGATCGAGGAGAGGCTTAAGACCTACGCCCATCTGTTCGACGATAAAGTGATGAAGCAACTTAAACGGAGAAGATATACCGGATGGGGACGCCTTAGCCGCAAACTCATCAACGGAATCCGGGACAAACAGAGCGGAAAGACCATTCTTGATTTCCTTAAGAGCGACGGATTCGCTAATCGCAACTTCATGCAACTTATCCATGATGATTCCCTGACCTTTAAGGAGGACATCCAGAAGGCCCAAGTGTCTGGACAAGGTGACTCACTGCACGAGCATATCGCAAATCTGGCTGGTTCACCCGCTATTAAGAAGGGTATTCTCCAGACCGTGAAAGTCGTGGACGAGCTGGTCAAGGTGATGGGTCGCCATAAACCAGAGAACATTGTCATCGAGATGGCCAGGGAAAACCAGACTACCCAGAAGGGACAGAAGAACAGCAGGGAGCGGATGAAAAGAATTGAGGAAGGGATTAAGGAGCTCGGGTCACAGATCCTTAAAGAGCACCCGGTGGAAAACACCCAGCTTCAGAATGAGAAGCTCTATCTGTACTACCTTCAAAATGGACGCGATATGTATGTGGACCAAGAGCTTGATATCAACAGGCTCTCAGACTACGACGTGGACGCCATCGTCCCTCAGAGCTTCCTCAAAGACGACTCAATTGACAATAAGGTGCTGACTCGCTCAGACAAGAACCGGGGAAAGTCAGATAACGTGCCCTCAGAGGAAGTCGTGAAAAAGATGAAGAACTATTGGCGCCAGCTTCTGAACGCAAAGCTGATCACTCAGCGGAAGTTCGACAATCTCACTAAGGCTGAGAGGGGCGGACTGAGCGAACTGGACAAAGCAGGATTCATTAAACGGCAACTTGTGGAGACTCGGCAGATTACTAAACATGTCGCCCAAATCCTTGACTCACGCATGAATACCAAGTACGACGAAAACGACAAACTTATCCGCGAGGTGAAGGTGATTACCCTGAAGTCCAAGCTGGTCAGCGATTTCAGAAAGGACTTTCAATTCTACAAAGTGCGGGAGATCAATAACTATCATCATGCTCATGACGCATATCTGAATGCCGTGGTGGGAACCGCCCTGATCAAGAAGTACCCAAAGCTGGAAAGCGAGTTCGTGTACGGAGACTACAAGGTCTACGACGTGCGCAAGATGATTGCCAAATCTGAGCAGGAGATCGGAAAGGCCACCGCAAAGTACTTCTTCTACAGCAACATCATGAATTTCTTCAAGACCGAAATCACCCTTGCAAACGGTGAGATCCGGAAGAGGCCGCTCATCGAGACTAATGGGGAGACTGGCGAAATCGTGTGGGACAAGGGCAGAGATTTCGCTACCGTGCGCAAAGTGCTTTCTATGCCTCAAGTGAACATCGTGAAGAAAACCGAGGTGCAAACCGGAGGCTTTTCTAAGGAATCAATCCTCCCCAAGCGCAACTCCGACAAGCTCATTGCAAGGAAGAAGGATTGGGACCCTAAGAAGTACGGCGGATTCGATTCACCAACTGTGGCTTATTCTGTCCTGGTCGTGGCTAAGGTGGAAAAAGGAAAGTCTAAGAAGCTCAAGAGCGTGAAGGAACTGCTGGGTATCACCATTATGGAGCGCAGCTCCTTCGAGAAGAACCCAATTGACTTTCTCGAAGCCAAAGGTTACAAGGAAGTCAAGAAGGACCTTATCATCAAGCTCCCAAAGTATAGCCTGTTCGAACTGGAGAATGGGCGGAAGCGGATGCTCGCCTCCGCTGGCGAACTTCAGAAGGGTAATGAGCTGGCTCTCCCCTCCAAGTACGTGAATTTCCTCTACCTTGCAAGCCATTACGAGAAGCTGAAGGGGAGCCCCGAGGACAACGAGCAAAAGCAACTGTTTGTGGAGCAGCATAAGCATTATCTGGACGAGATCATTGAGCAGATTTCCGAGTTTTCTAAACGCGTCATTCTCGCTGATGCCAACCTCGATAAAGTCCTTAGCGCATACAATAAGCACAGAGACAAACCAATTCGGGAGCAGGCTGAGAATATCATCCACCTGTTCACCCTCACCAATCTTGGTGCCCCTGCCGCATTCAAGTACTTCGACACCACCATCGACCGGAAACGCTATACCTCCACCAAAGAAGTGCTGGACGCCACCCTCATCCACCAGAGCATCACCGGACTTTACGAAACTCGGATTGACCTCTCACAGCTCGGAGGGGATGAGGGAGCTCCCAAGAAAAAGCGCAAGGTAGGTG**GGCGCGCCGCTAGCAGCGGAAGTACACCCGCA**ATGGAACTGCCCACCTGCAGCTGTCTTGATCGAGTTATACAAAAAGACAAAGGCCCATATTATACACACCTTGGGGCAGGACCAAGTGTTGCTGCTGTCAGGGAAATCATGGAGAATAGGTATGGTCAAAAAGGAAACGCAATAAGGATAGAAATAGTAGTGTACACCGGTAAAGAAGGGAAAAGCTCTCATGGGTGTCCAATTGCTAAGTGGGTTTTAAGAAGAAGCAGTGATGAAGAAAAAGTTCTTTGTTTGGTCCGGCAGCGTACAGGCCACCACTGTCCAACTGCTGTGATGGTGGTGCTCATCATGGTGTGGGATGGCATCCCTCTTCCAATGGCCGACCGGCTATACACAGAGCTCACAGAGAATCTAAAGTCATACAATGGGCACCCTACCGACAGAAGATGCACCCTCAATGAAAATCGTACCTGTACATGTCAAGGAATTGATCCAGAGACTTGTGGAGCTTCATTCTCTTTTGGCTGTTCATGGAGTATGTACTTTAATGGCTGTAAGTTTGGTAGAAGCCCAAGCCCCAGAAGATTTAGAATTGATCCAAGCTCTCCCTTACATGAAAAAAACCTTGAAGATAACTTACAGAGTTTGGCTACACGATTAGCTCCAATTTATAAGCAGTATGCTCCAGTAGCTTACCAAAATCAGGTGGAATATGAAAATGTTGCCCGAGAATGTCGGCTTGGCAGCAAGGAAGGTCGACCCTTCTCTGGGGTCACTGCTTGCCTGGACTTCTGTGCTCATCCCTACAGGGCCATTCACAACATGAATAATGGAAGCACTGTGGTTTGTACCTTAACTCGAGAAGATAACCGCTCTTTGGGTGTTATTCCTCAAGATGAGCAGCTCCATGTGCTACCTCTTTATAAGCTTTCAGACACAGATGAGTTTGGCTCCAAGGAAGGAATGGAAGCCAAGATCAAATCTGGGGCCATCGAGGTCCTGGCACCCCGCCGCAAAAAAAGAACGTGTTTCACTCAGCCTGTTCCCCGTTCTGGAAAGAAGAGGGCTGCGATGATGACAGAGGTTCTTGCACATAAGATAAGGGCAGTGGAAAAGAAACCTATTCCCCGAATCAAGCGGAAGAATAACTCAACAACAACAAACAACAGTAAGCCTTCGTCACTGCCAACCTTAGGGAGTAACACTGAGACCGTGCAACCTGAAGTAAAAAGTGAAACCGAACCCCATTTTATCTTAAAAAGTTCAGACAACACTAAAACTTATTCGCTGATGCCATCCGCTCCTCACCCAGTGAAAGAGGCATCTCCAGGCTTCTCCTGGTCCCCGAAGACTGCTTCAGCCACACCAGCTCCACTGAAGAATGACGCAACAGCCTCATGCGGGTTTTCAGAAAGAAGCAGCACTCCCCACTGTACGATGCCTTCGGGAAGACTCAGTGGTGCCAATGCTGCAGCTGCTGATGGCCCTGGCATTTCACAGCTTGGCGAAGTGGCTCCTCTCCCCACCCTGTCTGCTCCTGTGATGGAGCCCCTCATTAATTCTGAGCCTTCCACTGGTGTGACTGAGCCGCTAACGCCTCATCAGCCAAACCACCAGCCCTCCTTCCTCACCTCTCCTCAAGACCTTGCCTCTTCTCCAATGGAAGAAGATGAGCAGCATTCTGAAGCAGATGAGCCTCCATCAGACGAACCCCTATCTGATGACCCCCTGTCACCTGCTGAGGAGAAATTGCCCCACATTGATGAGTATTGGTCAGACAGTGAGCACATCTTTTTGGATGCAAATATTGGTGGGGTGGCCATCGCACCTGCTCACGGCTCGGTTTTGATTGAGTGTGCCCGGCGAGAGCTGCACGCTACCACTCCTGTTGAGCACCCCAACCGTAATCATCCAACCCGCCTCTCCCTTGTCTTTTACCAGCACAAAAACCTAAATAAGCCCCAACATGGTTTTGAACTAAACAAGATTAAGTTTGAGGCTAAAGAAGCTAAGAATAAGAAAATGAAGGCCTCAGAGCAAAAAGACCAGGCAGCTAATGAAGGTCCAGAACAGTCCTCTGAAGTAAATGAATTGAACCAAATTCCTTCTCATAAAGCATTAACATTAACCCATGACAATGTTGTCACCGTGTCCCCTTATGCTCTCACACACGTTGCGGGGCCCTATAACCATTGGGTCCCTGCAGGAA**GCGGAAGTACACCCGCA**ATGGTGAGCAAGGGCGAGGAGCTGTTCACCGGGGTGGTGCCCATCCTGGTCGAGCTGGACGGCGACGTAAACGGCCACAAGTTCAGCGTGTCCGGCGAGGGCGAGGGCGATGCCACCTACGGCAAGCTGACCCTGAAGTTCATCTGCACCACCGGCAAGCTGCCCGTGCCCTGGCCCACCCTCGTGACCACCCTGACCTACGGCGTGCAGTGCTTCAGCCGCTACCCCGACCACATGAAGCAGCACGACTTCTTCAAGTCCGCCATGCCCGAAGGCTACGTCCAGGAGCGCACCATCTTCTTCAAGGACGACGGCAACTACAAGACCCGCGCCGAGGTGAAGTTCGAGGGCGACACCCTGGTGAACCGCATCGAGCTGAAGGGCATCGACTTCAAGGAGGACGGCAACATCCTGGGGCACAAGCTGGAGTACAACTACAACAGCCACAACGTCTATATCATGGCCGACAAGCAGAAGAACGGCATCAAGGTGAACTTCAAGATCCGCCACAACATCGAGGACGGCAGCGTGCAGCTCGCCGACCACTACCAGCAGAACACCCCCATCGGCGACGGCCCCGTGCTGCTGCCCGACAACCACTACCTGAGCACCCAGTCCGCCCTGAGCAAAGACCCCAACGAGAAGCGCGATCACATGGTCCTGCTGGAGTTCGTGACCGCCGCCGGGATCACTCTCGGCATGGACGAGCTGTACAAGGCGGCCGCAGATCCAAAAAAGAAGAGAAAGGTAGATCCAAAAAAGAAGAGAAAGGTAGATCCAAAAAAGAAGAGAAAGGTAGATACGGCCGCA**GAACAAAAACTCATCTCAGAAGAGGATCTG**AATGGGGCCGCA**TAG**TCTAGAAGCTCGCTGATCAGCCTCGACTGTGCCTTCTAGTTGCCAGCCATCTGTTGTTTGCCCCTCCCCCGTGCCTTCCTTGACCCTGGAAGGTGCCACTCCCACTGTCCTTTCCTAATAAAATGAGGAAATTGCATCGCATTGTCTGAGTAGGTGTCATTCTATTCTGG

**Preparation of the dCas9- TET1CD (inactive) - EGFP fusion protein**

The fusion protein of dCas9-TET1CD (inactive)-EGFP was generated by sequentially assembling the coding sequences of the desired proteins using standard restriction enzyme digest and ligation method. The inserts were incorporated into the pShooter (pCMV/myc/nuc) mammalian expression vector (V821-20, Life Technologies). Prior to the incorporation of inserts, an adapter molecule was introduced in the multiple cloning sites (MCS) of the vector with NcoI and PstI flanking at the 5’ and 3’ end, respectively. The adapter molecule was generated by annealing equimolar concentration of primers UP and LP at 50°C for 10 min. The nucleotide sequence of UP and LP are as follows:

UP: 5’-CATGGATCCGAGGCGCGCCGCTAGCGGTACCCTGCA-3’

LP: 5’-GGGTACCGCTAGCGGCGCGCCTCGGATC-3’.

The adapter thus made was ligated to the vector after double digestion with NcoI and PstI. This adapter molecule introduced new restriction sites to the vector, including BamHI, AscI, NheI, KpnI, and SfbI. The source plasmids of inactive TET1CD (#49959 from Dr. Keith Joung group), dCas9 (#51023 from [Bo Huang](http://www.addgene.org/browse/pi/1925/) and [Stanley Qi](http://www.addgene.org/browse/pi/1487/) lab) and EGFP (#23027 from Dr. Andrea LeBlanc group) were obtained from the Addgene plasmid repository ([https://www.addgene.org](https://www.addgene.org/)). The inserts were PCR amplified using the primers, mentioned in **Supplementary Table-2.** PCR and cloning conditions were kept similar to the TET1-dCas9-EGFP fusion protein. The inactive TET1CD fusion proteins were also sequenced with the same sequencing primers, as mentioned in Supplementary Table-1. The full length nucleotide sequence of the fusion protein is available as **supplementary sequence-3.**

**Supplementary sequence-4:** Target *BRCA1* promoter sequence before bisulfite conversion. sgRNA binding sites to the *BRCA1* promoter, sgRNA-1 is highlighted in pink, sgRNA-2 in blue, sgRNA-3 in dark-yellow, and sgRNA-4 in green.

> hg19_dna range = chr17:41278463-41279225 5'pad=0 3'pad = 0 strand = + repeat Masking = none

AAGCTGGTAAGGAAGCAGCCTGGGTTAGCTAGGGGTGGGGTCACGTCACACTAAGAGGGTTTGGAGAAGTTCAAGGGAGGAATCCTGCAAAGAAGAGGGGCGACTTTTTCCGTGTCTCCGGACAGCTAATCGTTTTAGTGACAGGATGAGAGAGCCCTTCGTGTTCTGAGGGACCGAGTGGGCGAAAAGCGCCGGAGAGTTGGAGAGTCTGTGGTTCAGAATGCGAGGTGACAACGTGCTAGCAGCCCTCGCTCGCTCTCGGCGCCTCCTCGGCCTTGGCGTCCATTCTGGCCGTGCTGGAGGAGCCCTTCAGCCCGCCACTGCGCTGTGGGGGCCCCTCTCTGGGCTGGCCGAAGCCAGAGCCGGCTCCCTCTGCTTGCGGGGAAGTGTGGAGGGAGAGGCGGGTGTGGGAACTGGGGCTGCGCGCAGCGCTCGCCAGCCAGCGCGAGTTCCAGGTGGGCGCGGGCTCAGCGGGCCCCGCACCCCCGGCCCCGGGCAGTCAGGGGCCTAGCACCCGGGCCAGCAGCTGCAGAGGGTGCGCCGGGTCCCCCAGCACTGCCGGCCCGCCTGCACCCCGCTTGAATTCTCACCGGGCCCCAGCCGCCCTGCACAGGGCAAGGCTCAGGACCTGCAGCCCGCCATGCCCGAGCCCCCTCCCAACCCCTGTGAGCTCCAGCGTGGCCTGAGCCTCCCCGACGGGCACCGCCCCCTGCTCCTCAGCGCCCGGTCCCATCGACTGCCCAAGGGCTGAGAGGAGTGCAGG

**Supplementary sequence-5:** Target *BRCA1* promoter sequence after bisulfite conversion, considering all C have been converted to T. The changes in methylation at the selective CpG sites were observed, followed by the co-transfection with sgRNAs and dCas9 fusion protein. The sgRNAs and their target adjacent CpG sites, which have been sequenced, are highlighted in same color as the corresponding sgRNA. All other non-sequenced CpG sites are highlighted in yellow.

**Supplementary sequence-6:** Designed sgRNA-1 string sequence (in red color font) in PCR cassette with U6 promoter against the *BRCA1* gene promoter sequence.

GGCCTATTGGTTAAAAAATGAGCTGATTTAACAAAAATTTAACGCGAATTAATTAAGGTCGGGCAGGAAGAGGGCCTATTTCCCATGATTCCTTCATATTTGCATATACGATACAAGGCTGTTAGAGAGATAATTAGAATTAATTTGACTGTAAACACAAAGATATTAGTACAAAATACGTGACGTAGAAAGTAATAATTTCTTGGGTAGTTTGCAGTTTTAAAATTATGTTTTAAAATGGACTATCATATGCTTACCGTAACTTGAAAGTATTTCGATTTCTTGGCTTTATATATCTTGTGGAAAGGACGAAACACCGGGGGTCACGTCACACTAAGAGTTTTAGAGCTAGAAATAGCAAGTTAAAATAAGGCTAGTCCGTTATCAACTTGAAAAAGTGGCACCGAGTCGGTGCTTTTTTCTAGTATACCGTCGACCTCTAGCTAGAGCTTGGCGTAATCATGGTCATAGCTGTTTCCTGTGTGAAATTGTTATCCGCTC

**Supplementary sequence-7:** Designed sgRNA-2 string sequence (in red color font) in PCR cassette with U6 promoter against the *BRCA1* gene promoter sequence.

GGCCTATTGGTTAAAAAATGAGCTGATTTAACAAAAATTTAACGCGAATTAATTAAGGTCGGGCAGGAAGAGGGCCTATTTCCCATGATTCCTTCATATTTGCATATACGATACAAGGCTGTTAGAGAGATAATTAGAATTAATTTGACTGTAAACACAAAGATATTAGTACAAAATACGTGACGTAGAAAGTAATAATTTCTTGGGTAGTTTGCAGTTTTAAAATTATGTTTTAAAATGGACTATCATATGCTTACCGTAACTTGAAAGTATTTCGATTTCTTGGCTTTATATATCTTGTGGAAAGGACGAAACACCGGGCGTCCATTCTGGCCGTGCGTTTTAGAGCTAGAAATAGCAAGTTAAAATAAGGCTAGTCCGTTATCAACTTGAAAAAGTGGCACCGAGTCGGTGCTTTTTTCTAGTATACCGTCGACCTCTAGCTAGAGCTTGGCGTAATCATGGTCATAGCTGTTTCCTGTGTGAAATTGTTATCCGCTC

**Supplementary sequence-8:** Designed sgRNA-3 string sequence (in red color font) in PCR cassette with U6 promoter against the *BRCA1* gene promoter sequence.

GGCCTATTGGTTAAAAAATGAGCTGATTTAACAAAAATTTAACGCGAATTAATTAAGGTCGGGCAGGAAGAGGGCCTATTTCCCATGATTCCTTCATATTTGCATATACGATACAAGGCTGTTAGAGAGATAATTAGAATTAATTTGACTGTAAACACAAAGATATTAGTACAAAATACGTGACGTAGAAAGTAATAATTTCTTGGGTAGTTTGCAGTTTTAAAATTATGTTTTAAAATGGACTATCATATGCTTACCGTAACTTGAAAGTATTTCGATTTCTTGGCTTTATATATCTTGTGGAAAGGACGAAACACCGCTCAGCGGGCCCCGCACCCCGTTTTAGAGCTAGAAATAGCAAGTTAAAATAAGGCTAGTCCGTTATCAACTTGAAAAAGTGGCACCGAGTCGGTGCTTTTTTCTAGTATACCGTCGACCTCTAGCTAGAGCTTGGCGTAATCATGGTCATAGCTGTTTCCTGTGTGAAATTGTTATCCGCTC

**Supplementary sequence-9:** Designed sgRNA-4 string sequence (in red color font) in PCR cassette with U6 promoter against the *BRCA1* gene promoter sequence. The sgRNA-4 binds to the promoter in the reverse order.

GGCCTATTGGTTAAAAAATGAGCTGATTTAACAAAAATTTAACGCGAATTAATTAAGGTCGGGCAGGAAGAGGGCCTATTTCCCATGATTCCTTCATATTTGCATATACGATACAAGGCTGTTAGAGAGATAATTAGAATTAATTTGACTGTAAACACAAAGATATTAGTACAAAATACGTGACGTAGAAAGTAATAATTTCTTGGGTAGTTTGCAGTTTTAAAATTATGTTTTAAAATGGACTATCATATGCTTACCGTAACTTGAAAGTATTTCGATTTCTTGGCTTTATATATCTTGTGGAAAGGACGAAACACCGGGGCCCGGTGAGAATTCAAGGTTTTAGAGCTAGAAATAGCAAGTTAAAATAAGGCTAGTCCGTTATCAACTTGAAAAAGTGGCACCGAGTCGGTGCTTTTTTCTAGTATACCGTCGACCTCTAGCTAGAGCTTGGCGTAATCATGGTCATAGCTGTTTCCTGTGTGAAATTGTTATCCGCTC

**Fragment-1 (F1)**

AAGTTGGTAAGGAAGTAGTTTGGGTTAGTTAGGGGTGGGGTTACGTTATATTAAGAGGGTTTGGAGAAGTTTAAGGGAGGAATTTTGTAAAGAAGAGGGG**CG**ATTTTTTT**CG**TGTTTT**CG**GATAGTTAAT**CG**TTTTAGTGATAGGATGAGAGAGTTTTTCGTGTTTTGAGGGATCGAGTGGGCGAAAAGCGTCGGAGAGTTGGAGAGTTTGTGGTTTAGAATG**CG**AGGTGATAA**CG**TGTTAGTAGTTTT**CG**TT**CG**TTTT**CG**G**CG**TTTTTT**CG**GTTTTGGCGTTTATTTTGGTCGTGTTGGAGGAGTTTTTTAGTT**CG**TTATTG**CG**TTGTGGGGGTTTTTTTTTGGGTTGGT**CG**AAGTTAGAGT**CG**GTTTTTTTTGTTTG**CG**GGGA

**Fragment-2 (F2)**

AGTGTGGAGGGAGAGGCGGGTGTGGGAATTGGGGTTG**CGCG**TAG**CG**TT**CG**TTAGTTAG**CGCG**AGTTTTAGGTGGG**CGCG**GGTTTAG**CG**GGTTT**CG**TATTTT**CG**GTTT**CG**GGTAGTTAGGGGTTTAGTATT**CG**GGTTAGTAGTTGTAGAGGGTG**CG**T**CG**GGTTTTTTAGTATTGT**CG**GTT**CG**TTTGTATTTCGTTTGAATTTTTATCGGGTTTTAGT**CG**TTTTGTATAGGGTAAGGTTTAGGATTTGTAGTTCGTTATGTTCGAGTTTTTTTTTAATTTTTGTGAGTTTTAGCGTGGTTTGAGTTTTTTCGACGGGTATCGTTTTTTGTTTTTTAGCGTTCGGTTTTATCGATTGTTTAAGGGTTGAGAGGAGTGTAGG
